# Smoothed quantile residual life regression analysis with application to the Korea HIV/AIDS cohort study

**DOI:** 10.1186/s12874-024-02159-9

**Published:** 2024-02-17

**Authors:** Soo Min Kim, Yunsu Choi, Sangwook Kang, Korea HIV/AIDS cohort study

**Affiliations:** 1 Department of Applied Statistics, College of Commerce and Economics, Yonsei University, Seoul, Republic of Korea; 2 Department of Statistics and Data Science, College of Commerce and Economics, Yonsei University, Seoul, Republic of Korea; 3 Department of Preventive Medicine, College of Medicine, Hanyang University, Seoul, Republic of Korea; 4 Institute for Health and Society, College of Medicine, Hanyang University, Seoul, Republic of Korea; 5 Division of Infectious Disease, Department of Internal Medicine, Seoul St. Mary’s Hospital, College of Medicine, Catholic University, Seoul, Republic of Korea

**Keywords:** AIDS, Human immunodeficiency virus, Induced smoothing, Quantile regression, Residual life, Survival analysis

## Abstract

**Background:**

The residual life of a patient with human immunodeficiency virus (HIV) is of major interest to patients and their physicians. While existing analyses of HIV patient survival focus mostly on data collected at baseline, residual life analysis allows for dynamic analysis based on additional data collected over a period of time. As survival times typically exhibit a right-skewed distribution, the median provides a more useful summary of the underlying distribution than the mean. In this paper, we propose an efficient inference procedure that fits a semiparametric quantile regression model assessing the effect of longitudinal biomarkers on the residual life of HIV patients until the development of dyslipidemia, a disease becoming more prevalent among those with HIV.

**Methods:**

For estimation of model parameters, we propose an induced smoothing method that smooths nonsmooth estimating functions based on check functions. For variance estimation, we propose an efficient resampling-based estimator. The proposed estimators are theoretically justified. Simulation studies are used to evaluate their finite sample performances, including their prediction accuracy. We analyze the Korea HIV/AIDS cohort study data to examine the effects of CD4 (cluster of differentiation 4) cell count on the residual life of HIV patients to the onset of dyslipidemia.

**Results:**

The proposed estimator is shown to be consistent and normally distributed asymptotically. Under various simulation settings, our estimates are approximately unbiased. Their variances estimates are close to the empirical variances and their computational efficiency is superior to that of the nonsmooth counterparts. Two measures of prediction performance indicate that our method adequately reflects the dynamic character of longitudinal biomarkers and residual life. The analysis of the Korea HIV/AIDS cohort study data shows that CD4 cell count is positively associated with residual life to the onset of dyslipidemia but the effect is not statistically significant.

**Conclusions:**

Our method enables direct prediction of residual lifetimes with a dynamic feature that accommodates data accumulated at different times. Our estimator significantly improves computational efficiency in variance estimation compared to the existing nonsmooth estimator. Analysis of the HIV/AIDS cohort study data reveals dynamic effects of CD4 cell count on the residual life to the onset of dyslipidemia.

**Supplementary Information:**

The online version contains supplementary material available at 10.1186/s12874-024-02159-9.

## Background

The life expectancy of patients with human immunodeficiency virus (HIV) infection has increased considerably since antiretroviral therapy has become widespread. The life expectancy of HIV patients aged 20 years who began antiretroviral therapy between 2008 and 2010 was predicted to be approximately 78 years [1]. HIV patients live longer lives than before, increasing the number of non-acquired immunodeficiency syndrome (AIDS)-related morbidities [2]. Before 2007, AIDS was the leading cause of death in Korea. However, the rate of AIDS-related mortality has declined over time, while non-AIDS-related mortality has recently increased among patients with HIV in Korea [3, 4]. Research shows that compared to healthy individuals, patients with HIV are more likely to develop cardiovascular diseases, diabetes, hypertension, kidney diseases, liver diseases, psychological disorders, and various malignancies unrelated to AIDS [5]. Similar results were observed in Korea. Accordingly, from 2006 to 2016, syphilis, dyslipidemia, and cardiovascular disease were the most prevalent non-AIDS comorbidities among patients with HIV [4].

Our motivating dataset was obtained from a Korea HIV/AIDS cohort study aimed at preventing, treating, and effectively managing patients with HIV in Korea. The Korea HIV/AIDS cohort study is a multi-center cohort study that began in December 2006 and has thus far included over 1,500 participants [6]. As patients in the Korea HIV/AIDS cohort study lived longer, research on non-AIDS-related comorbidities became more critical. In this paper, we consider a time-to-event analysis of dyslipidemia, an increasingly common comorbidity among patients with HIV in Korea.

HIV patients who participated in the cohort study regularly visited hospitals for treatment. At each visit, information was obtained, including longitudinal biomarkers such as CD4 (cluster of differentiation 4) cell count. Since longitudinal biomarkers provide valuable insights into the clinical course of patients with HIV, assessing the impact of biomarkers is critical. The residual life regression model can immediately capture the effect of longitudinal biomarkers collected over time in the cohort data because it estimates the remaining lifetimes defined at various points until the event of interest. Studies based on residual life are naturally more dynamic than those based on existing popular survival models by updating new data at different follow-up time points.

In this paper, we propose a regression model of residual life to assess the effects of a longitudinal biomarker, CD4 cell count, an important biomarker for HIV patients. We allowed time-varying regression coefficients and time-varying covariates to capture the dynamic effects of CD4 cell count evaluated at different follow-up times. The distribution of survival time was typically skewed, with a long right tail. A mean survival time is not the best measure for summarizing the distribution. Quantiles, including the median, have become popular for describing the distribution of survival times. Thus, we suggest modeling the quantiles of residual life. Koenker and Bassett Jr [7] introduced the concept of quantile regression models. They presented a statistical approach for analyzing a semiparametric quantile regression model in the absence of censored data. Expanding upon their work, subsequent research delved into semi-parametric regression models for quantiles in the context of censored failure times [8–12]. Powell [13] proposed an inference method for the quantile regression model based on the least absolute deviation (LAD) principle, tailored particularly for censored data. Portnoy [10] introduced an approach that extends the Kaplan-Meier estimator to the realm of quantile regression. Please review and edit the paragraph accordingly. Peng and Huang [11] proposed a method based on the counting process and martingale framework that utilizes the Nelson-Aalen estimator of the cumulative hazard function. For flexibility, we consider a semiparametric quantile residual life regression model that does not assume a specific parametric distribution for survival times. Li et al. [14] and Lin et al. [15] proposed a statistical inference procedure for fitting this semiparametric quantile residual life regression model using time-varying biomarkers (e.g., BCR-ABL gene) as covariates with time-varying regression coefficients. They proposed estimating the regression coefficients using nonsmooth estimating functions with an L1-minimization algorithm. Although this algorithm is computationally efficient, the estimation of the variance of the estimated regression coefficients is based on a bootstrap method, which requires many calculations of the estimated regression coefficients. The variance estimation procedure is thus computationally intensive. Here, we propose an induced smoothing procedure [16] which has been shown to be more computationally efficient in situations considering semiparametric AFT models [17–19] and quantile regression models [20], especially in variance estimation. To the best of our knowledge, statistical methodologies that apply the induced smoothing method to fit semiparametric quantile residual life regression models exist only for models with time-invariant covariates and regression coefficients [21].

The remainder of this paper is organized as follows. Model and methods section introduces semiparametric quantile regression models and estimation methods based on the induced smoothing approach and establishes the asymptotic properties of the proposed estimators. Next, Simulation section presents the simulation studies that examine the finite sample properties of the proposed estimators. Lastly, Analysis of Korea HIV/AIDS cohort study data section applies the proposed methods to censored survival data from a Korea HIV/AIDS cohort study. In Supplementary material, we provide a sketch of the proofs of the asymptotic properties of our proposed estimators.

## Model and methods

### Semiparametric quantile regression model for longitudinal biomarkeres

Let *T* and *C* denote potential failure and censoring times, respectively. $Y=\min (T,C)$ denotes the observed time. We define the event indicator as $\delta =I(T \le C)$, where $I(\cdot )$ is the indicator function. $\varvec{X}$ denotes a set of vector of covariates, further divided into a subset of the time-invariant covariates ${\varvec{W}}=[1, W_1,\ldots ,W_p]^{\top } \in \mathbb {R}^p$ and a subset of the time-varying covariates ${\varvec{Z}(t)}=[Z_1(t),\ldots ,Z_q(t)]^{\top } \in \mathbb {R}^q$ where $\top$ denotes a transpose. To accommodate a missingness in $\varvec{Z}(t)$, we introduce an indicator for the *j*th visit $\eta _{j}, (j=1,2,\ldots ,D)$ where *D* denotes the planned visit time at $t_1< \ldots <t_D$. If $\varvec{Z}(t)$ is available at the *j*th planned visit, $\eta _{j}=1$. $\eta _{j}=0$, otherwise. The observed data are then made up of *n* independent copies of $\{Y,\delta ,\varvec{W},\eta , \eta _1\varvec{Z}(t_1),\ldots ,\eta _D\varvec{Z}(t_D)\}$, $\{Y_i,\delta _i,\varvec{W}_i,\eta _i, \eta _1\varvec{Z}(t_1),\ldots ,\eta _{iD}\varvec{Z}_i(t_D)\}^{n}_{i=1}$, where *n* and *i* stand for the sample size and subject, respectively.

The $\tau$th quantile of *T* is defined as the minimum time at which the cumulative distribution function (cdf) for *T* exceeds $\tau$ ($0< \tau < 1$). Specifically,

$$\begin{aligned} Q_{T}( \tau | \varvec{X}) = \inf \{t: F(t | \varvec{X}) \ge \tau \}, \quad 0< \tau < 1, \end{aligned}$$

where $F(t | \varvec{X}) = \Pr (T \le t | \varvec{X})$ denotes the cdf of *T* at time *t*.

Residual life is defined as the remaining period of life from time *t* to the event of interest. The residual life is denoted as $T-t$. We consider the following regression model for $Q_{T-t}( \tau | \varvec{X})$: To accommodate longitudinal biomarkers, we assume that $\varvec{X}$ is possibly time-dependent, i.e., $\varvec{X} = \varvec{X}(t)$, and can be divided into a set of time-varying covariates including longitudinal biomarkers, $\varvec{Z}(t)$, and time-invariant covariates, $\varvec{W}$, i.e., $\varvec{X}(t) = \{{\varvec{W}}^{\top }, \varvec{Z}(t)^{\top } \}^{\top }$. Then, the $\tau$th quantile residual life regression model [14] is

1 $$\begin{aligned} \log [Q_{T-t}(\tau | T\ge t, {\varvec{W}}, {\varvec{Z}(t)})]={\varvec{\alpha }(\tau , t)}^{\top } {\varvec{W}}+{\varvec{\beta }(\tau , t)}^{\top } {\varvec{Z}(t)} \end{aligned}$$

where ${\varvec{\alpha }}(\tau , t) = \{\alpha _{0}(\tau , t), \alpha _{1}(\tau , t), \ldots , \alpha _{p}(\tau , t)\}^{\top }$ and ${\varvec{\beta }}(\tau , t) = \{\beta _{1}(\tau , t), \ldots , \beta _{q}(\tau , t)\}^{\top }$ are $(p+1) \times 1$ and $q \times 1$ possibly time-varying regression coefficients for $\varvec{W}$ and $\varvec{Z}(t)$, respectively. Hereafter, whenever it is obvious, we suppress $\tau$ from ${\varvec{\alpha }}(\tau , t)$ and ${\varvec{\beta }}(\tau , t)$ for notational simplicity.

### Nonsmooth estimating functions

To estimate the time-varying regression coefficients $\varvec{\alpha }(t)$ and $\varvec{\beta }(t)$, we impose the restriction that $\varvec{\alpha }(t)$ and $\varvec{\beta }(t)$ can be expressed as linear combinations of finite basis functions. Specifically,

2 $$\begin{aligned} {\alpha _j}(t)&= \sum _{l=0}^{L}{{a_{j,l}}\times {f_l(t)}}, \quad j=0,1,..,p,\nonumber \\ {\beta _k}(t)&= \sum _{l=0}^{L}{b_{k,l}\times {f_l(t)}}, \quad k=1,..,q, \end{aligned}$$

where $f_0(t), f_1(t),\ldots , f_L(t)$ are predefined basis functions, and *L* is a finite positive integer. *B*-Spline basis and fractional polynomial basis functions [22, 23] are some popular choices. In Li et al. [14], fractional polynomial basis functions were considered.

Let $\varvec{\xi }(t)=\{f_0(t), f_1(t),\ldots , f_L(t)\}^{\top }$ and ${\varvec{U}(t)}=\{\varvec{\xi }(t), {\varvec{W}}\varvec{\xi }(t), {\varvec{Z}(t)}\varvec{\xi }(t)\}^{\top }$. Now, by taking advantage of these expression in (2), $\varvec{\alpha }(t)$ and $\varvec{\beta }(t)$, i.e., ${\alpha _j}(t) (j = 0, 1, \ldots , p)$ and ${\beta _k}(t) (k = 1, \ldots , q)$, can be estimated using the following estimating functions [14].

3 $$\begin{aligned} \varvec{S}(\varvec{\gamma };\tau )&=\frac{1}{n}\sum _{i=1}^{n}\varvec{S}_i(\varvec{\gamma },t;\tau )\nonumber \\&=\frac{1}{n}\sum _{i=1}^{n} I(Y_i>t){\varvec{U}_i(t)} \left\{ I(\log (Y_i-t)\le \varvec{\gamma }^{\top }{\varvec{U}_i(t)}) \frac{\delta _i}{{\hat{G}(Y_i)}/{\hat{G}(t)}}-\tau \right\} \end{aligned}$$

where $\varvec{\gamma }=(a_{0,0},a_{0,1},\ldots ,a_{0,L},\ldots ,a_{p,0},\ldots ,a_{p,L},b_{1,0},\ldots ,b_{1,L},\ldots ,b_{q,0},\ldots ,b_{q,L})^{\top }$, $\hat{G}(\cdot )$ is the estimated survival function for censoring times. $\varvec{U}_i$=$(\varvec{\xi }(t), X_{i1}\varvec{\xi }(t),\ldots ,X_{ip}\varvec{\xi }(t), Z_{i1}(t)\varvec{\xi }(t),\ldots ,Z_{iq}(t)\varvec{\xi }(t))^{\top }$, which is a matrix that combines the basis function and the subject *i*’s time-invariant and time-varying covariates.

Patients’ time-varying covariate ${\varvec{Z}(t)}$ are typically examined at several follow-up visits, and patients may not present at some follow-up visits, which would lead to missing values. (3) were further extended to accommodate these [14]. Specifically,

4 $$\begin{aligned} \dot{\varvec{S}}(\varvec{\gamma };\tau )&=\frac{1}{n}\sum _{i=1}^{n}\sum _{j=1}^{D}\eta _{ij}\varvec{S}_i(\varvec{\gamma },t_{ij};\tau )\\&=\frac{1}{n}\sum _{i=1}^{n}\sum _{j=1}^{D}\eta _{ij}I(Y_i>t_{ij})\varvec{U}_i(t_{ij})\left\{ I(\log (Y_i-t_{ij})\le \varvec{\gamma }^{\top }{\varvec{U}_i}(t_{ij}) \frac{\delta _i}{{\hat{G}(Y_i)}/{\hat{G}(t_{ij})}}-\tau \right\} \nonumber \end{aligned}$$

where $t_{ij}$ denotes the subject *i*’s *j*th visit time.

Solving (4) is equivalent to minimizing the objective function (5)

5 $$\begin{aligned} L(\varvec{\gamma }, \tau )&= n^{-1}\sum _{i=1}^n \sum _{j=1}^D w_{ij} \left| \log (Y_i - t_{ij}) - \varvec{\gamma }^{\top }\varvec{U}_i(t_{ij}) \right| \nonumber \\&\quad + \left| M - \varvec{\gamma }^{\top }n^{-1}\sum _{i=1}^n\sum _{j=1}^{n_i} - \varvec{U}_i(t_{ij})w_{ij}\right| + \left| M - \varvec{\gamma }^{\top }n^{-1}\sum _{i=1}^n\sum _{j=1}^{n_i} 2 \varvec{U}_i(t_{ij})\eta _{ij}I(Y_i>t_{ij})\right| \end{aligned}$$

where

$$\begin{aligned} w_{ij}=\frac{\eta _{ij}\delta _i I(Y_i>t_{ij})}{\hat{G}(Y_i)/\hat{G}(t_{ij})}, \end{aligned}$$

and *M* is an extremely large positive constant (e.g., $M = 10^6$). Existing software that can implement an L1-minimization algorithm, such as the $\mathbf {rq()}$ function in the quantreg package in R, can readily obtain $\hat{\varvec{\gamma }}$ [7]. The estimated $\alpha (t)$ and $\beta (t)$ can then be obtained by plugging in $\hat{\varvec{\gamma }}$ in (2).

### Induced smoothed estimating functions

Brown & Wang [16] proposed to use continuously differentiable functions to approximate discontinuous but monotone estimating functions via an induced smoothing method. We also propose to use the induced smoothed version of estimating functions (4) given by equations (6). Specifically,

6 $$\begin{aligned} \varvec{\tilde{S}}(\varvec{\gamma }; \tau ,\varvec{H})&\equiv E\left[ {\dot{\varvec{S}}(\varvec{\gamma }+\varvec{H}^{1/2}\varvec{Q}})\right] \nonumber \\&=\frac{1}{n}\sum _{i=1}^{n}\sum _{j=1}^{D}\eta _{ij}I(Y_i>t_{ij})\varvec{U}_i(t_{ij})\left\{ \Phi \left( \frac{\varvec{\gamma }^{\top }{\varvec{U}_i}(t_{ij})-\log (Y_i-t_{ij})}{\sqrt{\varvec{U}_i(t_{ij})^{\top }\varvec{H}{\varvec{U}_i(t_{ij})}}}\right) {\frac{\delta _i}{\hat{G}(Y_i)/\hat{G}(t_{ij})}}-\tau \right\} = 0, \end{aligned}$$

where $\varvec{Q}$ be an $N(\varvec{0},\varvec{I}_p$) random vector, $\varvec{I}_p$ represents for the $p\times p$ identity matrix. $\varvec{H}$ is a $p \times p$ symmetric and positive definite matrix, such that $\Vert \varvec{H} \Vert =O(n^{-1})$. $\Phi (\cdot )$ denotes the CDF of a standard normal distribution. $\tilde{\varvec{\gamma }}$, which is the induced smoothing estimator for $\varvec{\gamma }$, is defined as the solution to (6).

### Asymptotic properties

We summarize the asymptotic properties of the proposed induced smoothed estimator in the following theorem.

#### Theorem 1

Assuming the regularity conditions C1-C4 in the supplementary material hold, $\tilde{\varvec{\gamma }}$, solution to $\varvec{\tilde{S}}(\varvec{\gamma }) = 0$, is consistent for $\varvec{\gamma }_0$. $n^{1/2}(\tilde{\varvec{\gamma }}-{\varvec{\gamma }_0})$ converges to a zero-mean normal random variable. In addition, $n^{1/2}(\tilde{\varvec{\gamma }}-{\varvec{\gamma }_0})$ and $n^{1/2}(\hat{\varvec{\gamma }}-{\varvec{\gamma }_0})$ has the same asymptotic distribution where $\hat{\varvec{\gamma }}$ is the nonsmooth counterpart of $\tilde{\varvec{\gamma }}$ which minimizes (5).

Regularity conditions C1 - C4 and a proof of Theorem 1 are provided in Supplementary material. Due to the complicated nature of the asymptotic covariance function whose form is difficult to evaluate [14], we estimate it using an efficient resampling-based robust sandwich-type estimator. It is in the following Variance estimation section.

### Variance estimation

For variance estimation, Li et al. [14] proposed a resampling method that requires solving perturbed nonsmooth estimating equations or optimizing perturbed objective functions many times. This can be computationally demanding because a large number of parameters are often involved. On the other hand, the proposed induced smoothed estimating functions are continuously differentiable with respect to the regression parameters. This enables the use of a robust sandwich-form estimator, which is a common approach in variance estimation based on estimating equations.

We propose to employ a robust sandwich estimator $\hat{Var}(\tilde{\varvec{\gamma }})=\{\tilde{\varvec{A}}(\tilde{\varvec{\gamma }})^{-1}\}^{\top } \hat{\varvec{V}}(\tilde{\varvec{\gamma }})\{\tilde{\varvec{A}}(\tilde{\varvec{\gamma }})^{-1}\}$. The two components $\tilde{\varvec{A}}(\varvec{\tilde{\gamma} })$ and $\hat{\varvec{V}}(\tilde{\varvec{\gamma }})$ can be obtained separately. $\tilde{\varvec{A}}(\varvec{\tilde{\gamma} })$ is obtained by taking the first derivative of $\tilde{\varvec{S}}(\varvec{\gamma }; \tau ,\varvec{H})$ with respect to $\varvec{\gamma }$ evaluated at $\tilde{\varvec{\gamma }}$. Specifically,

$$\begin{aligned} \tilde{\varvec{A}}(\varvec{\gamma })&=\frac{{\partial {\varvec{\tilde{S}}(\varvec{\gamma }; \tau ,\varvec{H})}}}{\partial {\varvec{\gamma }}}\\&=\frac{1}{n}\sum _{i=1}^{n}\sum _{j=1}^{D}\eta _{ij}I(Y_i>t_{ij}){\frac{\delta _i}{\hat{G}(Y_i)/\hat{G}(t_{ij})}} \ \Phi \left( \frac{\varvec{\gamma }^{\top }{\varvec{U}_i}(t_{ij})-\log (Y_i-t_{ij})}{\sqrt{\varvec{U}_i(t_{ij})^{\top }\varvec{H}{\varvec{U}_i}(t_{ij})}}\right) {\frac{\varvec{U}_i(t_{ij})^{\top }{\varvec{U}_i(t_{ij})}}{\sqrt{\varvec{U}_i(t_{ij})^{\top }\varvec{H}{\varvec{U}_i(t_{ij})}}}}, \end{aligned}$$

where $\Phi (\cdot )$ denotes the cumulative distribution function of a standard normal random variable. $\hat{\varvec{V}}(\tilde{\varvec{\gamma }})$ can be obtained by using a computationally efficient resampling method. A similar approach was employed for the induced smoothed estimators under a semiparametric AFT model [20] and semiparametric quantile regression models for residual lifetimes [21]. First, we generate *n* independently and identically distributed weights ${\theta _i}$, $i=1,2, \ldots , n$ from an exponential distribution with a unit mean. Then, we construct $\varvec{\tilde{\varvec{S}}^{\star }}(\tilde{\varvec{\gamma }}; \tau , \varvec{H})$, a perturbed version of $\varvec{\tilde{\varvec{S}}}(\tilde{\varvec{\gamma }}; \tau , \varvec{H})$, using data with *n* realized values of ${\theta _i}$, where

$$\begin{aligned} \varvec{\tilde{\varvec{S}}^{\star }}(\tilde{\varvec{\gamma }}; \tau , \varvec{H})=\frac{1}{n}\sum _{i=1}^{n}\theta _i\sum _{j=1}^{D}\eta _{ij}I(Y_i>t_{ij})\varvec{U}_i(t_{ij})\left\{ \Phi \left( \frac{\varvec{\gamma }^{\top }{\varvec{U}_i}(t_{ij})-\log (Y_i-t_{ij} )}{\sqrt{\varvec{U}_i(t_{ij})^{\top }\varvec{H}{\varvec{U}_i}(t_{ij})}}\right) {\frac{\delta _i}{\hat{G}^{\star }(Y_i)/\hat{G}^{\star }(t_{ij})}}-\tau \right\} \end{aligned}$$

Note that $\hat{G}^{\star }(\cdot )$, a perturbed version of $\hat{G}(\cdot )$, should also be used. By repeating this procedure *K* times, we generate $\tilde{\varvec{S}}^{\star (1)}(\tilde{\varvec{\gamma }}; \tau , \varvec{H}), \cdots , \tilde{\varvec{S}}^{\star (K)}(\tilde{\varvec{\gamma }}; \tau , \varvec{H})$. $\hat{V}(\tilde{\varvec{\gamma }})$ is obtained using the sample variance of $\tilde{\varvec{S}}^{\star (1)}(\tilde{\varvec{\gamma }}; \tau , \varvec{H}), \cdots , \tilde{\varvec{S}}^{\star (K)}(\tilde{\varvec{\gamma }}; \tau , \varvec{H})$.

## Simulation

Extensive simulation experiments were conducted to evaluate the performance of the proposed induced smoothed estimators for finite samples. In addition, we compared the performance of our proposed estimators with that of Li et al. [14], a nonsmooth counterpart. The simulation settings considered are similar to those in Section 3 from Li et al. [14]. We denote our proposed method as “IS" and [14]’s method as “NS”, respectively.

### Simulation setup I

We first consider a simulation setting with a single time-invariant covariate and a single time-varying covariate; however, the time-varying covariate is non-informative; that is, the corresponding regression coefficient is set to zero. The time-invariant covariate *W* is generated from a Bernoulli distribution with a success probability of 0.5. The potential failure time *T* is generated from exponential distributions with means of 1 and 1.5 when $W=1$ and $W=1.5$, respectively. Censoring times were generated from $\kappa Unif(0,4)+4(1-\kappa )$ where $\kappa$ is a Bernoulli random variable with success probability 0.9. The time-varying covariate *Z*(*t*) is generated from a $Unif(-1,1)$ distribution at time *t*. We considered 12 planned visit times for each setup, and the visit times were $t = 0.1,0.15,0.2,0.25,0.3,0.4,0.5,0.6,0.7,0.8,0.9$, and 1.0. Given *t*, *W* and *Z*(*t*), the remaining lifetimes at *t* are then generated from the percentages of the following model: $\log [Q_{T-t}(\tau | T\ge t, {W}, {Z(t)})]=\alpha ^{\tau }_0(t)+\alpha ^{\tau }_1(t){W} + \beta ^{\tau }(t)Z(t)$. Because a patient can miss his/her visit, we generate a visit indicator for the *i*th patient at *j*th visit time, $\eta _{ij}=I(Y_i\ge t_j)\zeta _{ij}$ where $\zeta _{ij}$ follows the Bernoulli distribution with probability $p_V=p_{V0}I(W=0)+p_{V1}I(W=1)$. $p_V$ depends on *W* and $(p_{V0},p_{V1})$ is set to (0.75, 0.9).

The true regression coefficients of $\{\alpha ^{\tau }_0(t),\alpha ^{\tau }_1(t)\}$ are ($-1.65,0.41$) and $\beta ^{\tau }(t)=0$ at $\tau =0.25$. At $\tau =0.5$, the true regression coefficients of $\{\alpha ^{\tau }_0(t),\alpha ^{\tau }_1(t)\}$ are ($-0.77,0.41$), and the corresponding true regression coefficients of $\beta ^{\tau }(t)=0$. The sample size was set to 400. The average censoring proportion is $19\%$. To estimate the regression coefficients, we consider fractional polynomial basis functions $\varvec{\xi }(t)=\{1,\log (t),\sqrt{(}t),1/\sqrt{(}t)\}^{\top }$. $\alpha _0(t), \alpha _1(t)$ and $\beta (t)$ are estimated at four different time points: $t=0.1,0.2,0.5$ and 0.8. The resampling size for estimating the standard errors was set to 200. For each configuration, 1000 data sets are generated.

Table 1 displays the simulation results for Simulation setup I for the proposed induced smoothed estimators. For two values of $\tau$ and the corresponding true values of the parameters (TRUE), the empirical bias (EB), empirical standard error (ESE), and the average of the estimated standard error (ASE) for each combination of the setup were evaluated at four different time points. Overall, the results are satisfactory, and the proposed estimator is nearly unbiased. The proposed standard error estimates are in close agreement with their empirical counterparts in all settings considered.

**Table 1:** Summary of simulation results under Simulation setup I

				Induced smoothing method								
t	TRUE			EB			ESE			ASE		
	$\varvec{\alpha }_{\varvec{0}}\varvec{(t)}$	$\varvec{\alpha }_{\varvec{1}}\varvec{(t)}$	$\varvec{\beta (t)}$	$\varvec{\alpha }_{\varvec{0}}\varvec{(t)}$	$\varvec{\alpha }_{\varvec{1}}\varvec{(t)}$	$\varvec{\beta (t)}$	$\varvec{\alpha }_{\varvec{0}}\varvec{(t)}$	$\varvec{\alpha }_{\varvec{1}}\varvec{(t)}$	$\varvec{\beta (t)}$	$\varvec{\alpha }_{\varvec{0}}\varvec{(t)}$	$\varvec{\alpha }_{\varvec{1}}\varvec{(t)}$	$\varvec{\beta (t)}$
$$\tau =0.25$$												
0.1	-1.65	0.41	0.00	0.001	0.001	-0.006	0.177	0.236	0.198	0.171	0.237	0.198
0.2	-1.65	0.41	0.00	-0.007	0.010	-0.001	0.160	0.219	0.130	0.165	0.232	0.132
0.5	-1.65	0.41	0.00	0.001	-0.003	0.005	0.168	0.226	0.143	0.163	0.229	0.146
0.8	-1.65	0.41	0.00	0.002	-0.003	0.008	0.187	0.250	0.148	0.177	0.248	0.164
$$\tau =0.5$$												
0.1	-0.77	0.41	0.00	0.007	-0.005	-0.006	0.130	0.173	0.151	0.127	0.176	0.148
0.2	-0.77	0.41	0.00	-0.006	-0.003	0.001	0.135	0.180	0.095	0.131	0.184	0.099
0.5	-0.77	0.41	0.00	-0.001	-0.002	0.002	0.154	0.210	0.109	0.151	0.210	0.112
0.8	-0.77	0.41	0.00	-0.006	0.003	0.003	0.182	0.244	0.113	0.166	0.231	0.122

### Simulation setup II

For this setup, we considered an informative time-varying covariate, and the corresponding regression coefficient was nonzero. The time-invariant covariate *W* and censoring time *C* were generated in the same manner as in Simulation setup I. *T* is generated from a $Weibull(\lambda ,2)$ distribution. Note that, under the $Weibull(\lambda ,2)$ distribution, it can be shown that the quantile residual life function at given *t* and $\tau$ equals $\sqrt{-\log (1-\tau )/\lambda +t^2}-t$ [24]. In this setup, $\lambda$ is allowed to vary and is generated from the *Unif*(0.5, 1.5) distribution. The time-varying covariate *Z*(*t*) for a given $t,\tau$ and $\lambda$ are ${Z}(t)=\log [\sqrt{\{}-\log (1-\tau )/(\lambda t^2)+1\}-1]/\sqrt{t}$ and the corresponding remaining lifetimes are generated from $\log [Q_{T-t}(\tau | T\ge t, {W}, Z(t))]=\log (t)+\sqrt{t}{Z}(t)$. For the probability of visiting $p_V=p_{V0}I(W=0)+p_{V1}I(W=1)$ in the Bernoulli distribution, which models the visiting probability of a patient at a specific time point, we set $(p_{V0},p_{V1})$ to (0.5, 0.7). The average censoring proportion is $21\%$. For comparison, we also calculated the nonsmooth estimator by Li et al. [14]. For each combination, the process is repeated 1000 times.

**Fig. 1:**
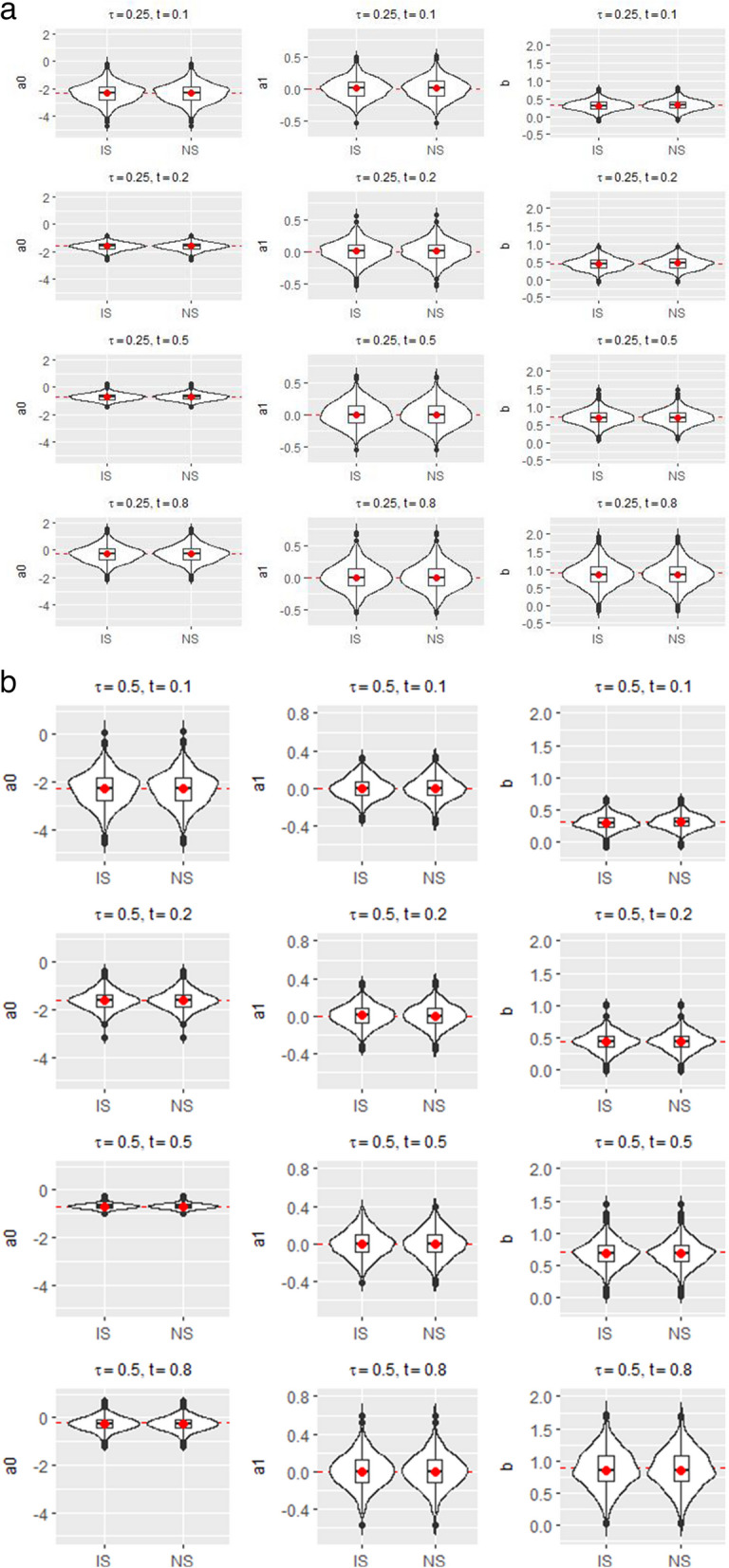
Violin plots of estimated regression coefficients by two methods under simulation setup II. IS and NS denote the proposed induced smoothed estimator and nonsmooth estimator, respectively: **a** $\tau =0.25$; **b** $\tau =0.5$

The results are summarized in Fig. 1. The violin plots in Fig. 1 compare the estimates of the proposed induced smoothed method (IS) and those of the nonsmooth counterpart (NS). The red dotted line in each plot represents the true regression coefficient values for given *t* and $\tau$. In general, both estimators produced similar results. For $\tau =0.5$, both estimators exhibit negligible biases. Although variabilities vary for different *t* values, the magnitudes of variabilities of the two estimators are similar. The same conclusion was drawn for $\tau =0.25$. The proposed standard error estimates are close to their empirical counterparts (see Table S1 in Supplementary material).

We also compared the performances of IS and NS in terms of the computational speed for calculating variance estimates. We considered the median ($\tau =0.5$) at $t=0.5$ for varying sample sizes of $n=200, 400$ and 800 in Simulation setup II. This comparison was performed on a 2.30 GHz Intel(R) Quad Core(TM) i7-11800H central processing unit (CPU) using R 4.3.2. [25]. The results are summarized in Table 2. Our proposed variance estimator is $10 \sim 20$ times faster than its nonsmooth counterpart, which reveals its superiority in computational efficiency, especially in variance estimation.

**Table 2:** Summary of runtimes (in seconds) of estimation under Simulation setup II

	Method	
*n*	NS	IS
200	22.4	1.9
400	84.9	4.6
800	341.8	13.9

### Simulation setup III

We also consider the case where the structural form of the regression coefficients in (2) is misspecified. In Li et al. [14], we use a similar setting to test the robustness of our method against misspecification. We modify Simulation Setup II by setting the time-varying covariate *Z*(*t*) as follows: $Z(t)={\frac{\log [\sqrt{-\log (1-\tau )/(\lambda t^2)+1}-1]}{0.1(t+1)^2+0.1/t}}$. The corresponding remaining lifetime at *t* is $\log [Q_{T-t}(\tau | T\ge t, W, Z(t)] = \log (t)+\left\{ 0.1(t+1)^2+0.1/t\right\} {Z}(t)$.

Table 3 summarizes the estimates based on the proposed induced smoothing method. The results demonstrate that the proposed estimator works reasonably well and produces negligible bias. The ESEs and ASEs were generally in good agreement with each other. We also conducted a sensitivity analysis to check whether the estimation results were affected by a different choice of basis function. Two different sets of basis functions for fractional polynomial basis, $\varvec{\xi }(t)=\{1,\log (t),\sqrt{(}t),1/\sqrt{(}t),1/t\}^{\top }$ and $\varvec{\xi }(t)=\{1,1/\sqrt{(}t),t,t^2\}^{\top }$, and a *B*-spline basis. We consider a *B*-spline basis with zero, one, and two knots. The results are presented in Additional results of simulation studies of Supplementary material (Tables S2 - S6). They are comparable to those obtained using the basis functions considered throughout the simulation experiments, $\varvec{\xi }(t)=\{1,\log (t),\sqrt{(}t),1/\sqrt{(}t)\}^{\top }$. This result implies that the estimated coefficients are robust to the choice of basis functions. Furthermore, we also considered increased censoring rates of 24% in Simulation setup I and 28% in Simulation setups II and III. The performance of the proposed method remained largely similar, with slightly increased standard errors. The results are provided in the Additional results of simulation studies of Supplementary material (Tables S7 - S9).

**Table 3:** Summary of simulation results under simulation setup III

				Induced smoothing method								
t	TRUE			EB			ESE			ASE		
	$\varvec{\alpha }_{\varvec{0}}\varvec{(t)}$	$\varvec{\alpha }_{\varvec{1}}\varvec{(t)}$	$\varvec{\beta (t)}$	$\varvec{\alpha }_{\varvec{0}}\varvec{(t)}$	$\varvec{\alpha }_{\varvec{1}}\varvec{(t)}$	$\varvec{\beta (t)}$	$\varvec{\alpha }_{\varvec{0}}\varvec{(t)}$	$\varvec{\alpha }_{\varvec{1}}\varvec{(t)}$	$\varvec{\beta (t)}$	$\varvec{\alpha }_{\varvec{0}}\varvec{(t)}$	$\varvec{\alpha }_{\varvec{1}}\varvec{(t)}$	$\varvec{\beta (t)}$
$$\tau =0.5$$												
0.1	-2.30	0.00	1.12	-0.053	-0.005	0.036	0.676	0.113	0.371	0.623	0.112	0.342
0.2	-1.61	0.00	0.64	-0.004	0.003	-0.001	0.383	0.111	0.199	0.363	0.109	0.187
0.5	-0.69	0.00	0.43	0.003	0.002	-0.010	0.107	0.143	0.117	0.102	0.142	0.112
0.8	-0.22	0.00	0.45	-0.033	0.006	-0.014	0.276	0.173	0.150	0.265	0.169	0.148

### Prediction

By modeling the residual lifetimes at different time points with repeatedly measured longitudinal biomarkers and time-varying coefficients, the residual lifetimes can be predicted dynamically [15]. To assess the prediction performance of the proposed method, we considered two measures representing two essential aspects of prediction accuracy: calibration and discrimination. The first is the absolute loss between the predicted residual lifetime and corresponding true value [15]. This absolute loss is a measure of calibration and is referred to as $MAE_p$ and is defined as follows:

$$\begin{aligned} \text {E}|\min \{T_i - t, (L-t)\}-Q_i^{p}| T_i>t|, \end{aligned}$$

where $T_i - t$ is the true residual life for subject *i* at time *t*, which is always available in the simulation experiments; $Q_i^{p}$ is the predicted $\tau$th residual lifetime for subject *i* and *L* is a constant that truncates the residual lifetimes. The second is the concordance index (C-index) [26]. The C-index measures discrimination and quantifies the proportion of correctly ordered pairs of predicted survival times. Following Lin et al. [15], who adapted the C-index from Uno et al. [27], we use the C-index defined as

$$\begin{aligned} \hat{C_L}(t)=\frac{\sum _{i=1}^{n}\sum _{j=1}^{n}\delta _i\{\hat{G}(Y_i)/\hat{G}(t)\}^{-2}I(0< Y_i - t< Y_j - t,Y_i<L) I(\hat{Y}_{i} - t< \hat{Y}_j - t)}{\sum _{i=1}^{n}\sum _{j=1}^{n}\delta _i\{\hat{G}(Y_i)/\hat{G}(t)\}^{-2}I(0< Y_i - t< Y_j - t, Y_i < L)}, \end{aligned}$$

where $\hat{Y}_i - t$ is the predicted residual life for subject *i* at prediction time *t*. For both measures, we restrict our attention to the observed times in (0, *L*) where *L* is slightly shorter than the maximum censoring time. As pointed out in Lin et al. [15], $MAE_p$ is a measure of calibration, whereas the C-index is a measure of discrimination. We calculated $MAE_p$ values and C-indices based on our proposed induced smoothed and nonsmooth estimator [14].

**Fig. 2:**
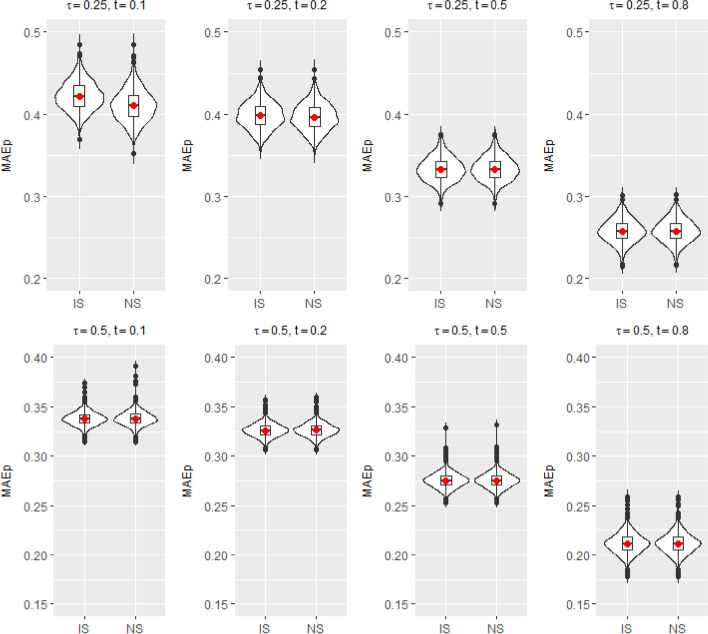
$MAE_p$s between predicted residual life and the true values based on the proposed induced smooth method (IS) and nonsmooth method (NS) under Simulation setup II for $\tau = 0.25, 0.5$ and $t = 0.1, 0.2, 0.5$ and 0.8

**Fig. 3:**
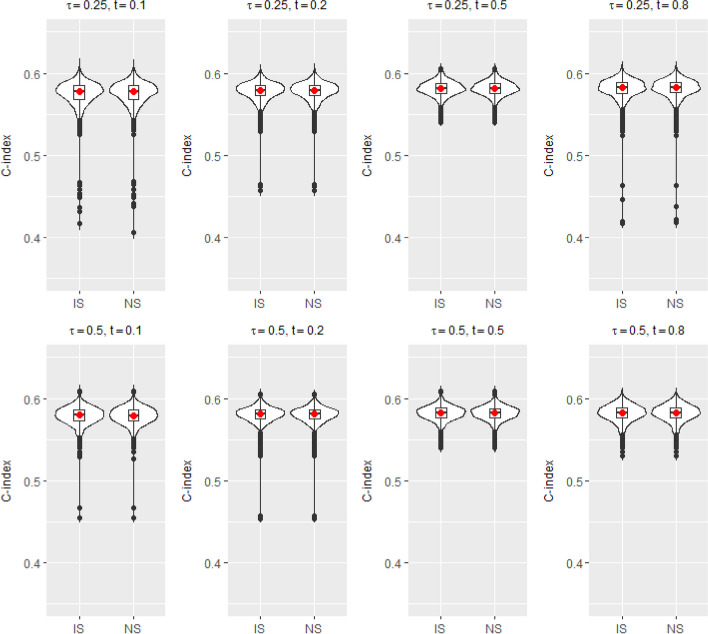
C-indices between predicted residual life and the true values based on the proposed induced smooth method (IS) and nonsmooth method (NS) under Simulation setup II for $\tau = 0.25, 0.5$ and $t = 0.1, 0.2, 0.5$ and 0.8

The data were generated based on the setup described in Simulation Setup II. Training and test datasets were generated independently. The sample sizes for the training and test data sets are 400 and 10, 000, respectively. To calculate the predicted residual lifetime, we used the estimated regression coefficients obtained from the training dataset for the covariates in the test dataset. We consider two quantiles: $\tau =0.25$ and $\tau =0.5$. To calculate $MAE_p$s and C-indices, we set *L* as the $5^{th}$ percentile of the test dataset’s censoring time, close to the maximum censoring time. We repeatedly computed $MAE_p$ values and C-indices 1, 000 times. Figures 2 and 3 show violin plots for $MAE_p$s and C-indices, respectively, based on 1, 000 simulations of simulation setup II for two different quantiles and four different prediction times ($t=0.1, 0.2, 0.5$ and 0.8). Figure 2 shows that as the prediction time points increase, $MAE_p$ decreases. This pattern reflects the dynamic prediction aspect by utilizing the accumulated information over time. Meanwhile, the C-indices were fairly consistent across the different prediction time points, in the range of $0.57 - 0.58$ (Fig. 3).

## Analysis of Korea HIV/AIDS cohort study data

We applied our proposed method to the Korea HIV/AIDS cohort study data. HIV patients live longer than ever before, resulting in an increase in the incidence of deaths from non-AIDS complications, particularly dyslipidemia, a major risk factor for coronary artery disease and stroke [2, 28–30]. Indeed, as patients with HIV began to receive long-term, highly active antiretroviral therapy, reports of long- and short-term adverse effects of these treatments began to emerge. Several studies have shown that HIV patients receiving long-term antiretroviral therapy develop metabolic disorders such as dyslipidemia, insulin resistance, glucose intolerance, metabolic bone disease, and lactic acidosis [31–35]. Thus, our application aims to model the remaining lifetime until the incidence of dyslipidemia in patients with HIV in the Korea HIV/AIDS cohort study data.

We analyze data from male patients with 1486 total HIV patients who participated in the survey before 2012 in the Korea HIV/AIDS cohort study. Follow-ups began on the date of HIV diagnosis. As mentioned above, the event of interest was the onset of dyslipidemia after an HIV diagnosis. Since diabetes and high blood pressure are risk factors for dyslipidemia, participants who developed diabetes, high blood pressure, or dyslipidemia before being diagnosed with HIV were excluded from the study. Patients with missing age data at diagnosis were excluded. The resulting dataset comprised 502 patients. A patient’s survival time was considered censored if they died before experiencing dyslipidemia or had no recorded dyslipidemia during the last clinic visit. The results revealed that 163 patients (32.5 %) had dyslipidemia.

**Fig. 4:**
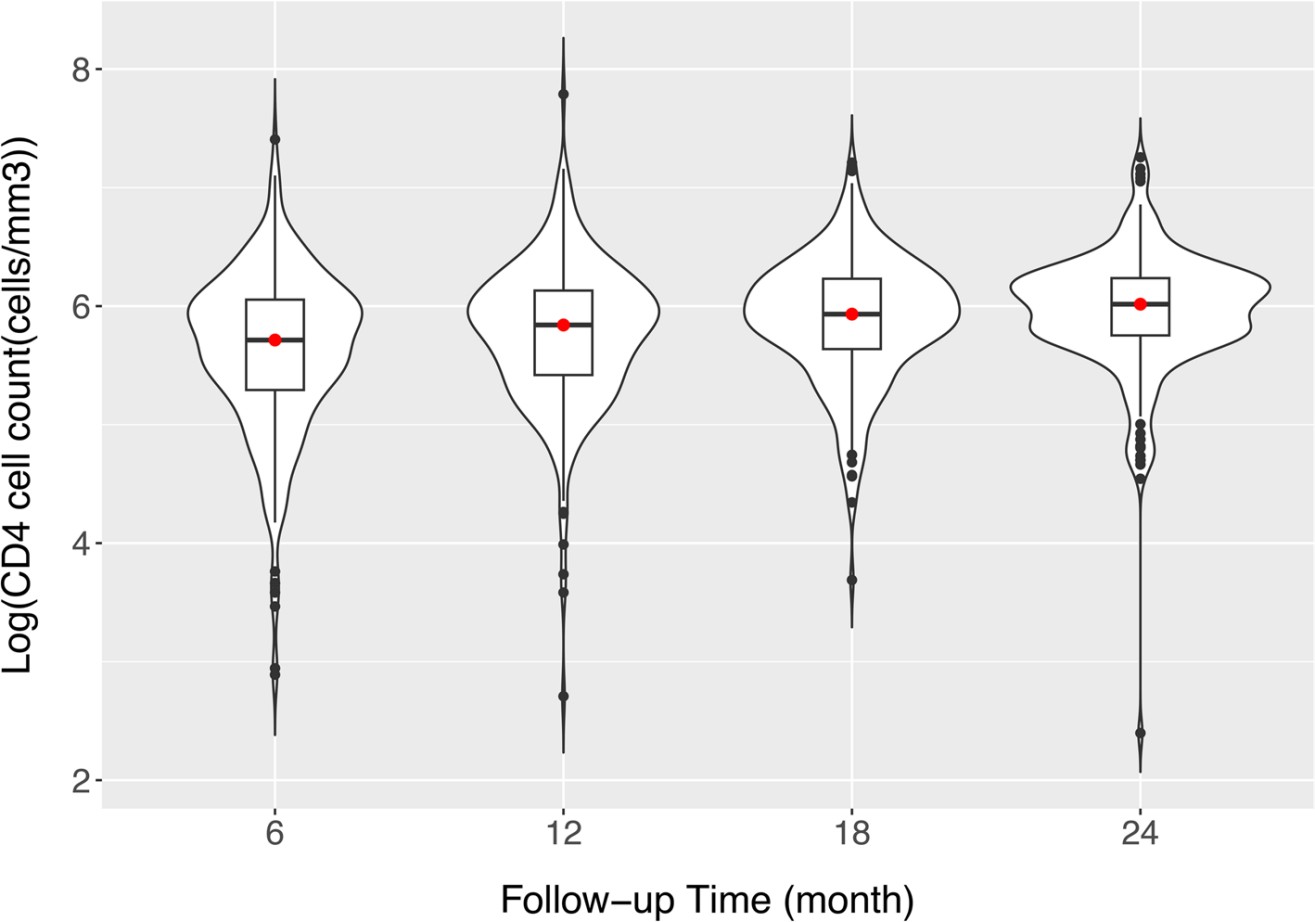
Summary of the Korea HIV/AIDS cohort study data - violin plot of $\log$ of CD4 cell count ($cells/mm^3$) during follow-up visits until 24 months

In the Korea HIV/AIDS cohort study, CD4 cell count, an important immunological biomarker, was collected regularly. Patients with HIV who participated in the cohort study were surveyed every six months. CD4 cell counts provide information on the onset of dyslipidemia in patients with HIV. Several studies have found significant associations between dyslipidemia and CD4 cell counts in HIV-infected individuals [36, 37]. We used the (log-transformed) CD4 cell count as a time-varying covariate and assessed its effect on the time to onset of dyslipidemia. We evaluated these effects every six months for two years (at 6, 12, 18, and 24 months) by defining the remaining lifetimes at each time point. Because not every patient visits a hospital, every six months exactly, we used CD4 cell count measured within a two months interval for each time point if it was not measured every six months. Patient data with missing CD4 cell counts at all visits were excluded from the analysis. Figure 4 summarizes CD4 cell counts at 6, 12, 18, and 24-month follow-up visits. CD4 cell counts seemed to increase gradually over time, showing that the patient’s immunological state was improving. Notably, this phenomenon occurred when most cohort study participants received HIV treatment. Dyslipidemia is also strongly linked to age [38]. Women aged 45 and older and men aged 35 and older should be checked for dyslipidemia regularly [28]. Therefore, we dichotomized the age at HIV diagnosis as above or below 35 years and added it to our model as a time-fixed covariate. Furthermore, we used the (log-transformed) total cholesterol, high-density lipoprotein (HDL), and a family history of dyslipidemia as a time-fixed covariate that are related to the onset of dyslipidemia . Higher total cholesterol levels, lower HDL levels, and a family history of dyslipidemia are associated with an increased risk of the onset of dyslipidemia [39–42]. HDL, total cholesterol values and a family history of dyslipidemia at the time of diagnosis were not available in the Korean HIV/AIDS cohort data; instead, values from the initial survey were used. This is to adjust for the effect of CD4 cell count on dyslipidemia onset. Further, we assume that the relationship between the residual life of dyslipidemia onset and CD4 cell count, while adjusting for age at baseline, follows the quantile residual life regression model in (1). We fitted this model using the proposed weighted estimation equation approach. The standard error of the regression coefficient estimate was calculated using the resampling procedure described in Asymptotic properties section.

Before constructing the weighted estimation equations, we tested the assumption of independence between censoring times and covariates in the model by fitting a Cox regression model for censoring times, with CD4 cell count and age at diagnosis as covariates. The effects of the CD4 cell count, total cholesterol, HDL, family history of dyslipidemia and age at diagnosis were not statistically significant, with the corresponding p-values of 0.8094, 0.4984, 0.1672, 0.0675 and 0.4987, respectively. Thus, the Kaplan-Meier estimator, based on marginal censoring times, is utilized when constructing the weight function.

**Fig. 5:**
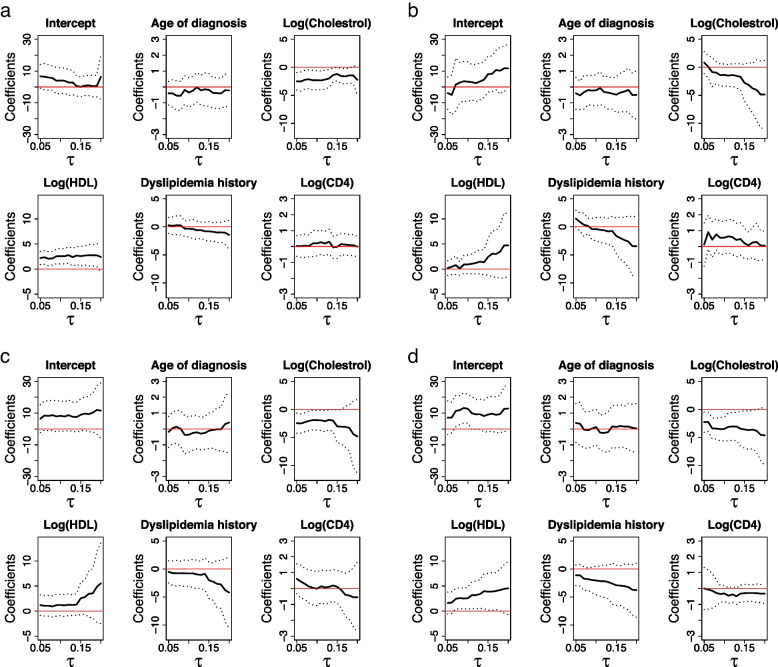
Plots of estimated regression coefficients (  ), and corresponding 95% point-wise confidence intervals (  ) for the Korea HIV/AIDS cohort study data evaluated for different quantiles ($\tau = 0.05 \sim 0.2$) at **a** 6 months; **b** 12 months; **c** 18 months; **d** 24 months

The results of the data analysis are summarized in Tables 4 and 5 and illustrated in Fig. 5. Tables 4 and 5 display the estimated regression coefficients evaluated at months 6, 12, 18, and 24 after dyslipidemia onset for quantiles in the range of $5\%$ to $20\%$ ($\tau = 0.05 - 0.20$), with the associated standard error estimates and 95% confidence intervals. Figure 5 shows the estimated regression coefficients for different quantiles considering the $\tau$s at different visit times, along with their point-wise 95% confidence intervals. Here, we focus on the lower quantiles owing to the identifiability issues induced by the high censoring rate. The estimated regression coefficients for CD4 cell counts were mostly positive for the quantiles and base times considered, implying that higher CD4 cell counts are associated with longer residual lifetimes to the onset of dyslipidemia in most cases. These effects, however, were not statistically significant at the significance level of 0.05. The total cholesterol level is negatively associated with the quantiles of residual lifetimes. When $\tau =0.15$ and $t=6$, for example, the estimated regression coefficient of the total cholesterol level is $-1.20$ with the corresponding standard error estimate of 0.61, statistically significant at the significance level of 0.05. This implies that for the 15th percentile of the residual lifetime evaluated six months after the baseline, a one-unit increase in total cholesterol level in the log-scale is estimated to decrease the corresponding quantile of the residual lifetime on the log scale by 1.20 months. The HDL level seem to be positively associated with the quantiles of residual lifetimes. Some of the effects were statistically significant at early evaluation times (6 months for $\tau = 0.05 \sim 0.15$). The results showed that the patients with the family history of dyslipidemia and aged 35 years or older at the time of diagnosis developed dyslipidemia more quickly than those without the family history and under 35, respectively. These effects were, however, not statistically significant. Similar results are shown in Fig. 5.

**Table 4:** Summary of analysis results of Korea HIV/AIDS cohort study. PE is the point estimate of the regression parameter. SE is the estimated standard error of the regression parameter. $95\%$ CI is the Wald-type $95\%$ point-wise confidence interval

		Induced smoothing method								
	Follow-up time (month)	Intercept			Age of diagnosis			CD4 cell count		
		PE	SE	95% CI	PE	SE	95% CI	PE	SE	95% CI
$\tau$=0.05										
	6	6.64	3.81	(-0.828, 14.105)	-0.40	0.38	(-1.144, 0.354)	0.03	0.33	(-0.621, 0.678)
	12	-3.90	5.21	(-14.103, 6.307)	-0.40	0.51	(-1.404, 0.608)	0.13	0.69	(-1.232, 1.485)
	18	6.53	4.46	(-2.215, 15.266)	-0.18	0.50	(-1.157, 0.793)	0.61	0.48	(-0.324, 1.553)
	24	7.08	5.40	(-3.507, 17.664)	0.38	0.62	(-0.835, 1.592)	0.00	0.68	(-1.327, 1.325)
$\tau$=0.1										
	6	4.07	4.17	(-4.108, 12.246)	-0.35	0.47	(-1.282, 0.576)	0.20	0.40	(-0.589, 0.988)
	12	3.29	6.39	(-9.230, 15.813)	-0.21	0.50	(-1.189, 0.775)	0.56	0.52	(-0.466, 1.577)
	18	8.31	4.85	(-1.187, 17.813)	-0.36	0.57	(-1.467, 0.749)	-0.01	0.54	(-1.072, 1.057)
	24	12.70	4.59	(3.706, 21.685)	0.12	0.57	(-0.990, 1.236)	-0.31	0.26	(-0.811, 0.200)
$\tau$=0.15										
	6	0.04	3.16	(-6.165, 6.236)	-0.19	0.53	(-1.224, 0.841)	-0.02	0.37	(-0.748, 0.716)
	12	8.03	5.77	(-3.289, 19.345)	-0.49	0.57	(-1.605, 0.631)	0.20	0.49	(-0.765, 1.160)
	18	8.95	5.14	(-1.134, 19.025)	-0.11	0.57	(-1.234, 1.012)	0.11	0.52	(-0.912, 1.129)
	24	9.08	5.58	(-1.846, 20.009)	0.13	0.68	(-1.203, 1.471)	-0.29	0.28	(-0.841, 0.269)
$\tau$=0.2										
	6	6.34	7.24	(-7.844, 20.529)	-0.22	0.55	(-1.308, 0.864)	-0.01	0.32	(-0.643, 0.630)
	12	11.70	7.68	(-3.346, 26.742)	-0.50	0.78	(-2.037, 1.029)	0.06	0.46	(-0.846, 0.969)
	18	11.57	8.89	(-5.866, 28.999)	0.41	0.99	(-1.521, 2.343)	-0.55	1.14	(-2.782, 1.674)
	24	12.87	7.83	(-2.482, 28.215)	0.03	0.80	(-1.543, 1.603)	-0.32	0.31	(-0.935, 0.299)

**Table 5:** Summary of analysis results of Korea HIV/AIDS cohort study. PE is the point estimate of the regression parameter. SE is the estimated standard error of the regression parameter. $95\%$ CI is the Wald-type $95\%$ point-wise confidence interval

		Induced smoothing method								
	Follow-up time (month)	Total cholesterol			HDL			Family history of dyslipidemia		
		PE	SE	95% CI	PE	SE	95% CI	PE	SE	95% CI
$\tau$=0.05										
	6	-2.50	0.79	(-4.052, -0.943)	2.21	0.66	(0.908, 3.510)	0.28	0.73	(-1.139, 1.708)
	12	0.83	1.00	(-1.132, 2.791)	0.21	0.75	(-1.247, 1.675)	1.47	0.76	(-0.008, 2.952)
	18	-2.46	0.91	(-4.251, -0.668)	1.20	1.04	(-0.832, 3.240)	-0.52	1.01	(-2.504, 1.454)
	24	-2.27	0.92	(-4.079, -0.466)	1.62	1.11	(-0.548, 3.794)	-1.14	0.94	(-2.979, 0.694)
$\tau$=0.1										
	6	-2.31	0.86	(-4.005, -0.624)	2.57	0.79	(1.016, 4.128)	-0.33	0.76	(-1.808, 1.157)
	12	-1.35	1.00	(-3.312, 0.605)	0.92	0.95	(-0.947, 2.792)	-0.39	1.05	(-2.451, 1.665)
	18	-1.93	0.90	(-3.691, -0.161)	1.22	1.08	(-0.896, 3.344)	-0.80	1.18	(-3.107, 1.509)
	24	-3.49	1.07	(-5.591, -1.387)	2.58	1.13	(0.372, 4.784)	-2.06	1.31	(-4.633, 0.516)
$\tau$=0.15										
	6	-1.20	0.61	(-2.389, -0.004)	2.57	1.02	(0.574, 4.572)	-0.80	0.88	(-2.535, 0.927)
	12	-2.77	1.65	(-6.007, 0.475)	2.39	1.84	(-1.217, 5.996)	-1.61	1.61	(-4.764, 1.547)
	18	-3.03	1.51	(-5.997, -0.063)	2.55	1.65	(-0.688, 5.784)	-2.03	1.65	(-5.264, 1.202)
	24	-3.58	1.71	(-6.925, -0.236)	3.88	1.86	(0.228, 7.528)	-2.92	1.84	(-6.520, 0.682)
$\tau$=0.2										
	6	-2.22	1.38	(-4.928, 0.497)	2.40	1.51	(-0.559, 5.368)	-1.43	1.33	(-4.031, 1.175)
	12	-4.84	3.09	(-10.891, 1.211)	4.72	3.21	(-1.576, 11.013)	-3.46	2.76	(-8.869, 1.954)
	18	-4.82	3.37	(-11.419, 1.782)	5.54	4.11	(-2.503, 13.589)	-4.21	3.23	(-10.543, 2.119)
	24	-4.64	2.74	(-10.013, 0.735)	4.55	2.69	(-0.718, 9.827)	-3.78	2.47	(-8.616, 1.060)

**Fig. 6:**
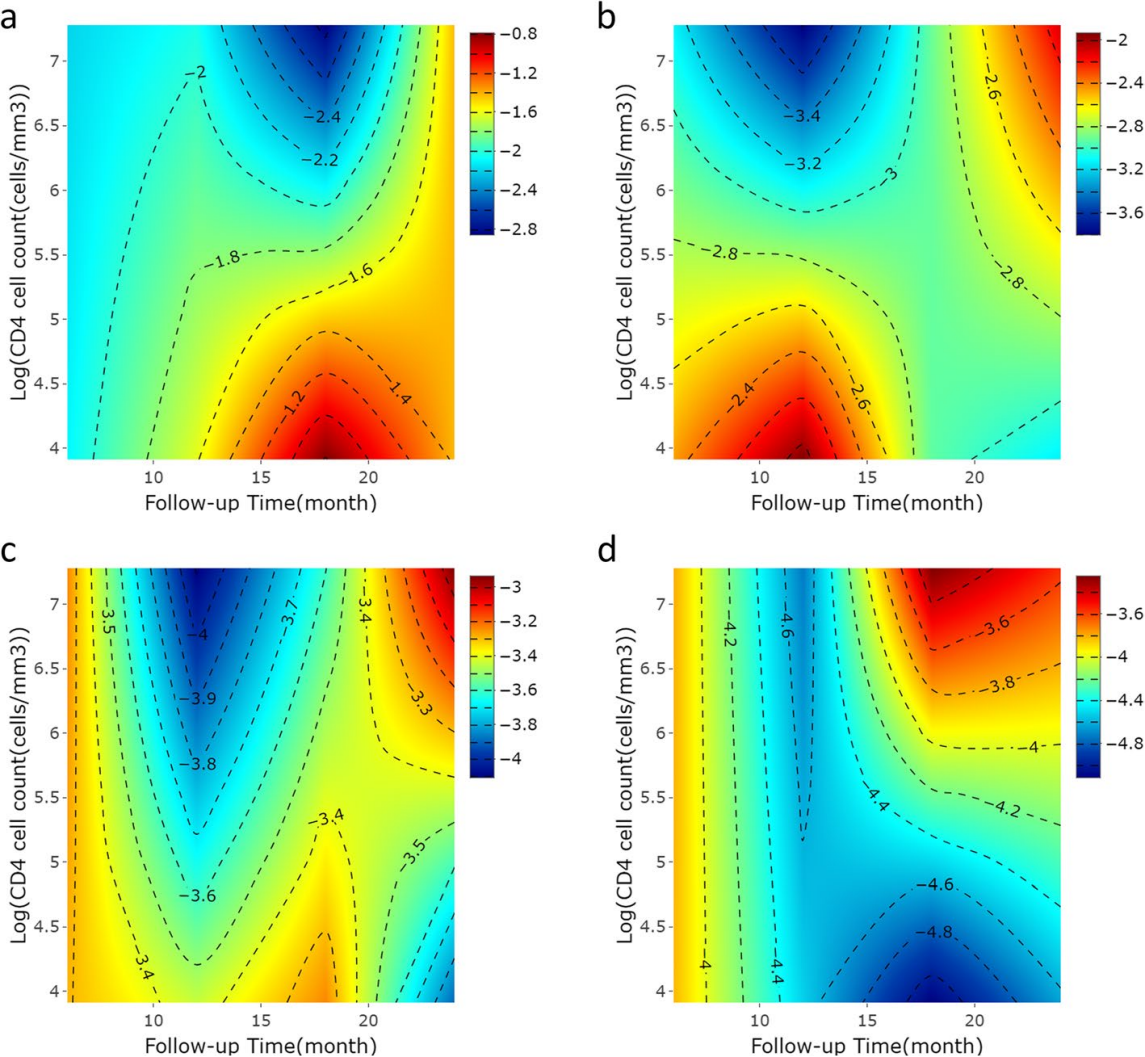
Estimated quantile residual life of HIV patients over 35 years old at diagnosis by follow-up time *t* and $\log$ CD4 cell count at time *t* where the color represents the estimated $\tau$th-quantile residual life (months) (  , contour curves): **a** $\tau =0.05$; **b** $\tau =0.1$; **c** $\tau =0.15$; **d** $\tau =0.2$

Based on the fitted model, we constructed a heat map in Fig. 6 that displays the predicted residual life until the dyslipidemia onset at different quantiles for those who are younger than 35 years old, have no family history of dyslipidemia, and have the average values of the log-transformed total cholesterol levels (5.10) and log-transformed HDL values (3.66). For different follow-up times (horizontal axis) and CD4 cell counts (vertical axis), the predicted residual lifetimes are plotted in different colors. The scale on the right indicates that red and blue represent shorter and longer anticipated residual lifetimes, respectively. For example, Fig. 6d shows that if the log-transformed CD4 cell count at six months of follow-up for a patient under 35 years of age at the time of diagnosis with the average values of 5.10 for the total cholesterol level and 3.66 for the HDL value, and without a family history was 6.11, the probability that the patient will develop dyslipidemia in the next 8.1 months is 5%. In addition, a patient with an decreased log-transformed CD4 cell count of 5.59 at 12 months of follow-up with the same values of age at diagnosis, total cholesterol level, HDL value and family history, had a 5% probability of developing dyslipidemia in 6.6 months, which is approximately 2 months shorter. In the Korea HIV/AIDS cohort study, patients whose CD4 cell count on the log-scale was 6.11 with the values of 5.24 for the total cholesterol level, 3.78 for HDL and without a family history at 6 months of follow-up had a residual lifetime until dyslipidemia onset of 8 months (2.08 on the log-scale), and patients whose CD4 cell count was 5.59, 5.16 for total cholesterol, 3.74 for HDL and without a family history at 12 months had a residual lifetime of 3 months (1.10 on the log-scale). Both are close to the predicted residual lifetimes of 8.1 and 6.6 months, respectively.

## Discussion

This study proposes applying an induced smoothing method to the existing nonsmooth estimator [14] to fit the semiparametric quantile residual life regression model for data with time-varying biomarkers that are repeatedly measured, such as CD4 cell counts in the Korea HIV/AIDS cohort study. The proposed induced smoothed estimator shares asymptotic normality and consistency with its nonsmooth counterpart while demonstrating its superiority in terms of computational efficiency via simulation experiments, especially in variance estimation. We implemented our proposed method to analyze Korea HIV/AIDS cohort study data. By modeling quantiles for residual lifetimes to the onset of dyslipidemia and applying our proposed induced smoothing method, we dynamically assessed the effect of CD4 cell count, a longitudinal biomarker, for different evaluation times and quantiles. In addition, a direct prediction of the residual lifetimes can be made, which also has a dynamic feature that accommodates the data accumulated at different evaluation times.

Caution should be exercised when applying the proposed method. Before using the weight - the inverse of the estimated censoring survival function - in (6), it is essential to check that the censoring survival function is marginal; that is, censoring times are independent of the covariates used in the model. The weight can be modified to allow dependence on covariates. In this case, the current method of estimating the censoring survival function using the Kaplan-Meier method can be replaced by a sensible regression model, such as a Cox model. The relevant part of deriving the asymptotic properties should also accommodate this change. Another limitation of our method is that the longitudinal biomarker measurements must be performed at specific intervals. One way to alleviate this restriction is to adopt the method proposed by Lin et al. [15], in which irregularly measured longitudinal biomarker measurements can be accommodated by extracting the dominant features over a certain period using a functional principal component analysis approach. Subsequent studies should consider this as a direction for future research.

In the simulation experiments and data analysis, we estimated conditional quantiles at several different quantile levels. Estimating quantiles separately, however, could lead to crossing quantiles, which does not guarantee the monotonicity of quantiles. To handle this issue, when dealing with a completely observed response variable, joint modeling of multiple quantiles [43–47] or implementing a second stage adjustment [48–50] have been proposed. The literature on censored quantile regression models has relative been limited. Tang and Wang [51] developed a joint modeling approach with a penalization procedure based on adaptive lasso. Yuan et al. [52] extended it to the fused adaptive lasso penalization. These methods are, however, based on modeling regular failure time *T*. No statistical methods have yet been developed to account for the correct ordering of estimated quantiles for residual lifetimes, even with time-invariant covariates and regression coefficients. Therefore, it would be an interesting future work extending the proposed work based on residual lifetimes to ensure the monotonicity of estimated conditional quantiles.

In the analysis of the Korean HIV/AIDS cohort study, we considered a model predicting residual life until the onset of dyslipidemia based on information regarding total cholesterol levels, high-density lipoprotein level, family history of dyslipidemia, age of diagnosis, and CD4 levels. However, there are other factors, such as smoking, frequent alcohol consumption, an unhealthy diet, and the use of protease inhibitors (PI), known to be associated with the onset of dyslipidemia [28, 53]. Unfortunately, these variables were either not incorporated into the data collection of the Korean HIV/AIDS cohort study, or if they were, the assessment intervals did not correspond with our study, and the response rates were too low to consider for analysis. Thus, it should be acknowledged that predicting residual life solely based on the variables used in real-data analysis may be less realistic. We wished to include them, but there were limitations in the available data. Nonetheless, the methodology provided in this study presents a general approach that can be applied to any dataset, allowing for the prediction of residual life until any event while accounting for any time-varying covariate. The identification of this potential demonstrates the proposed method’s adaptability. Given the availability of data, it is possible to use this method to predict residual life in a variety of scenarios.

### Supplementary Information

**Additional file 1.** Additional results of simulation studies.

## Data Availability

The data that support the findings of this study were obtained under license from the Korea Disease Control and Prevention Agency and are not publicly available due to restrictions. Access to the data may be granted by the author, Korea HIV/AIDS Cohort Study, upon request, subject to obtaining permission from the Korea Disease Control and Prevention Agency.

## Abbreviations

AIDS: acquired immunodeficiency syndrome
HIV: human immunodeficiency virus
