# Supplementary material for: Smoothed quantile residual life regression analysis with application to the Korea HIV/AIDS cohort study
Source: BMC Med Res Methodol. 2024 Feb 17;24:44. doi: 10.1186/s12874-024-02159-9 (PMC10873972; doi:10.1186/s12874-024-02159-9)
Supplement: Supplementary file 1 — Additional file 1. Additional results of simulation studies. [file 12874_2024_2159_MOESM1_ESM.pdf]

Additional results of simulation studies

Table S1 Summary of the simulation results under Simulation setup II

t	TRUE			Induced smoothing method								
				EB			ESE			ASE		
	$\alpha_0(t)$	$\alpha_1(t)$	$\beta(t)$	$\alpha_0(t)$	$\alpha_1(t)$	$\beta(t)$	$\alpha_0(t)$	$\alpha_1(t)$	$\beta(t)$	$\alpha_0(t)$	$\alpha_1(t)$	$\beta(t)$
$\tau = 0.25$												
0.1	-2.30	0.00	0.32	0.019	0.000	0.007	0.686	0.156	0.138	0.625	0.141	0.127
0.2	-1.61	0.00	0.45	-0.004	0.002	-0.004	0.269	0.158	0.165	0.271	0.162	0.164
0.5	-0.69	0.00	0.71	-0.011	0.006	-0.013	0.250	0.188	0.203	0.241	0.183	0.201
0.8	-0.22	0.00	0.89	-0.048	0.007	-0.026	0.586	0.198	0.333	0.558	0.196	0.318
$\tau = 0.5$												
0.1	-2.30	0.00	0.32	-0.001	-0.006	0.006	0.676	0.111	0.105	0.616	0.106	0.096
0.2	-1.61	0.00	0.45	0.009	0.004	-0.007	0.391	0.109	0.141	0.366	0.108	0.131
0.5	-0.69	0.00	0.71	0.002	0.002	-0.020	0.108	0.142	0.195	0.104	0.143	0.186
0.8	-0.22	0.00	0.89	-0.026	0.006	-0.026	0.279	0.174	0.300	0.267	0.168	0.293

Table S2 Summary of simulation results using polynomial basis $(1, \log(t), \sqrt(t), 1/\sqrt(t), 1/t)$ under Simulation setup II

t	TRUE			Induced smoothing method								
				EB			ESE			ASE		
	$\alpha_0(t)$	$\alpha_1(t)$	$\beta(t)$	$\alpha_0(t)$	$\alpha_1(t)$	$\beta(t)$	$\alpha_0(t)$	$\alpha_1(t)$	$\beta(t)$	$\alpha_0(t)$	$\alpha_1(t)$	$\beta(t)$
$\tau = 0.25$												
0.1	-2.30	0.00	0.32	0.007	0.002	0.049	0.653	0.150	0.132	0.556	0.130	0.113
0.2	-1.61	0.00	0.45	0.018	-0.001	-0.024	0.277	0.148	0.172	0.273	0.155	0.167
0.5	-0.69	0.00	0.71	-0.005	0.003	-0.011	0.298	0.197	0.255	0.285	0.190	0.249
0.8	-0.22	0.00	0.89	-0.043	0.005	-0.022	0.595	0.208	0.336	0.568	0.206	0.321
$\tau = 0.5$												
0.1	-2.30	0.00	0.32	-0.008	-0.005	0.015	0.711	0.115	0.111	0.723	0.121	0.113
0.2	-1.61	0.00	0.45	0.004	0.003	-0.008	0.374	0.106	0.135	0.344	0.103	0.123
0.5	-0.69	0.00	0.71	0.000	0.000	-0.024	0.108	0.146	0.222	0.106	0.148	0.217
0.8	-0.22	0.00	0.89	-0.024	0.004	-0.026	0.282	0.174	0.305	0.268	0.171	0.293

Table S3 Summary of simulation results using polynomial basis $(1, 1/\sqrt{(t)}, t, t^2)$ under Simulation setup II

t	TRUE			Induced smoothing method EB			ESE			ASE		
	$\alpha_0(t)$	$\alpha_1(t)$	$\beta(t)$	$\alpha_0(t)$	$\alpha_1(t)$	$\beta(t)$	$\alpha_0(t)$	$\alpha_1(t)$	$\beta(t)$	$\alpha_0(t)$	$\alpha_1(t)$	$\beta(t)$
$\tau = 0.25$												
0.1	-2.30	0.00	0.32	0.036	0.001	0.000	0.714	0.156	0.144	0.677	0.144	0.137
0.2	-1.61	0.00	0.45	0.002	0.001	-0.007	0.245	0.163	0.149	0.246	0.164	0.147
0.5	-0.69	0.00	0.71	-0.005	0.005	-0.007	0.276	0.191	0.230	0.264	0.183	0.228
0.8	-0.22	0.00	0.89	-0.040	0.007	-0.021	0.579	0.201	0.327	0.556	0.200	0.314
$\tau = 0.5$												
0.1	-2.30	0.00	0.32	0.035	-0.006	-0.001	0.680	0.109	0.105	0.630	0.105	0.098
0.2	-1.61	0.00	0.45	0.020	0.004	-0.011	0.365	0.111	0.131	0.337	0.110	0.120
0.5	-0.69	0.00	0.71	0.001	0.002	-0.018	0.106	0.144	0.211	0.102	0.145	0.201
0.8	-0.22	0.00	0.89	-0.024	0.005	-0.022	0.279	0.173	0.300	0.266	0.169	0.287

Table S4 Summary of simulation results using a B-spline basis (knot=null) in Simulation setup II

t	TRUE			Induced smoothing method EB			ESE			ASE		
	$\alpha_0(t)$	$\alpha_1(t)$	$\beta(t)$	$\alpha_0(t)$	$\alpha_1(t)$	$\beta(t)$	$\alpha_0(t)$	$\alpha_1(t)$	$\beta(t)$	$\alpha_0(t)$	$\alpha_1(t)$	$\beta(t)$
$\tau = 0.25$												
0.1	-2.30	0.00	0.32	0.115	0.000	0.024	0.621	0.158	0.127	0.633	0.161	0.129
0.2	-1.61	0.00	0.45	-0.012	0.002	0.007	0.256	0.155	0.159	0.258	0.153	0.159
0.5	-0.69	0.00	0.71	-0.011	0.005	-0.008	0.286	0.180	0.245	0.268	0.169	0.242
0.8	-0.22	0.00	0.89	-0.098	0.006	-0.056	0.595	0.228	0.332	0.563	0.222	0.318
$\tau = 0.5$												
0.1	-2.30	0.00	0.32	-0.160	-0.006	0.026	0.593	0.106	0.092	0.607	0.111	0.094
0.2	-1.61	0.00	0.45	-0.012	0.004	0.002	0.367	0.110	0.133	0.341	0.107	0.122
0.5	-0.69	0.00	0.71	-0.004	0.002	-0.018	0.105	0.141	0.221	0.097	0.137	0.213
0.8	-0.22	0.00	0.89	-0.049	0.005	-0.058	0.280	0.185	0.297	0.256	0.176	0.278

Table S5 Summary of simulation results using a B-spline basis (knot = 0.5) in Simulation setup II

t	TRUE			Induced smoothing method								
				EB			ESE			ASE		
	$\alpha_0(t)$	$\alpha_1(t)$	$\beta(t)$	$\alpha_0(t)$	$\alpha_1(t)$	$\beta(t)$	$\alpha_0(t)$	$\alpha_1(t)$	$\beta(t)$	$\alpha_0(t)$	$\alpha_1(t)$	$\beta(t)$
$\tau = 0.25$												
0.1	-2.30	0.00	0.32	-0.057	0.000	0.012	0.625	0.158	0.126	0.659	0.158	0.134
0.2	-1.61	0.00	0.45	-0.012	0.000	0.009	0.261	0.159	0.162	0.261	0.161	0.159
0.5	-0.69	0.00	0.71	-0.036	0.008	-0.027	0.310	0.222	0.261	0.296	0.212	0.254
0.8	-0.22	0.00	0.89	-0.038	0.001	-0.022	0.711	0.277	0.401	0.691	0.265	0.399
$\tau = 0.5$												
0.1	-2.30	0.00	0.32	-0.097	-0.005	0.016	0.618	0.110	0.096	0.627	0.113	0.097
0.2	-1.61	0.00	0.45	-0.016	0.004	0.005	0.368	0.112	0.133	0.358	0.115	0.128
0.5	-0.69	0.00	0.71	-0.006	0.001	-0.033	0.117	0.154	0.222	0.111	0.155	0.210
0.8	-0.22	0.00	0.89	-0.022	0.006	-0.017	0.306	0.208	0.333	0.294	0.202	0.325

Table S6 Summary of simulation results using a B-spline basis (knot = $(0.4, 0.7)$) in Simulation setup II

t	TRUE			Induced smoothing method								
				EB			ESE			ASE		
	$\alpha_0(t)$	$\alpha_1(t)$	$\beta(t)$	$\alpha_0(t)$	$\alpha_1(t)$	$\beta(t)$	$\alpha_0(t)$	$\alpha_1(t)$	$\beta(t)$	$\alpha_0(t)$	$\alpha_1(t)$	$\beta(t)$
$\tau = 0.25$												
0.1	-2.30	0.00	0.32	-0.010	-0.001	0.003	0.628	0.162	0.127	0.670	0.160	0.136
0.2	-1.61	0.00	0.45	-0.010	0.001	0.007	0.269	0.157	0.163	0.263	0.161	0.160
0.5	-0.69	0.00	0.71	-0.027	0.008	-0.023	0.317	0.210	0.272	0.292	0.201	0.263
0.8	-0.22	0.00	0.89	-0.071	-0.003	-0.040	0.789	0.280	0.443	0.743	0.267	0.420
$\tau = 0.5$												
0.1	-2.30	0.00	0.32	-0.062	-0.005	0.011	0.639	0.113	0.099	0.637	0.114	0.099
0.2	-1.61	0.00	0.45	-0.012	0.004	0.004	0.369	0.113	0.133	0.363	0.118	0.129
0.5	-0.69	0.00	0.71	-0.001	0.002	-0.027	0.114	0.151	0.229	0.103	0.143	0.215
0.8	-0.22	0.00	0.89	-0.029	0.006	-0.022	0.329	0.211	0.360	0.308	0.198	0.334

Table S7 Summary of simulation results under Simulation setup I with censoring rate= 24%

t	TRUE			Induced smoothing method								
				EB			ESE			ASE		
	$\alpha_0(t)$	$\alpha_1(t)$	$\beta(t)$	$\alpha_0(t)$	$\alpha_1(t)$	$\beta(t)$	$\alpha_0(t)$	$\alpha_1(t)$	$\beta(t)$	$\alpha_0(t)$	$\alpha_1(t)$	$\beta(t)$
$\tau = 0.25$												
0.1	-1.65	0.41	0.00	0.003	0.000	-0.003	0.177	0.237	0.201	0.174	0.240	0.199
0.2	-1.65	0.41	0.00	-0.013	0.019	0.001	0.163	0.226	0.133	0.165	0.234	0.133
0.5	-1.65	0.41	0.00	0.001	0.000	0.002	0.173	0.235	0.147	0.167	0.233	0.151
0.8	-1.65	0.41	0.00	-0.001	-0.002	0.008	0.194	0.262	0.158	0.180	0.255	0.169
$\tau = 0.5$												
0.1	-0.77	0.41	0.00	0.006	-0.005	-0.005	0.132	0.179	0.157	0.130	0.181	0.152
0.2	-0.77	0.41	0.00	-0.007	0.002	-0.001	0.138	0.189	0.100	0.133	0.188	0.103
0.5	-0.77	0.41	0.00	-0.004	0.006	0.002	0.160	0.217	0.115	0.155	0.219	0.120
0.8	-0.77	0.41	0.00	-0.005	0.005	0.000	0.191	0.256	0.125	0.179	0.254	0.134

Table S8 Summary of simulation results under Simulation setup II with censoring rate= 28%

t	TRUE			Induced smoothing method								
				EB			ESE			ASE		
	$\alpha_0(t)$	$\alpha_1(t)$	$\beta(t)$	$\alpha_0(t)$	$\alpha_1(t)$	$\beta(t)$	$\alpha_0(t)$	$\alpha_1(t)$	$\beta(t)$	$\alpha_0(t)$	$\alpha_1(t)$	$\beta(t)$
$\tau = 0.25$												
0.1	-2.30	0.00	0.32	0.020	0.000	0.007	0.696	0.157	0.141	0.637	0.144	0.129
0.2	-1.61	0.00	0.45	-0.003	0.000	-0.004	0.276	0.161	0.169	0.276	0.165	0.168
0.5	-0.69	0.00	0.71	-0.010	0.004	-0.013	0.256	0.192	0.204	0.249	0.188	0.207
0.8	-0.22	0.00	0.89	-0.046	0.004	-0.025	0.605	0.212	0.344	0.588	0.207	0.335
$\tau = 0.5$												
0.1	-2.30	0.00	0.32	0.000	-0.005	0.006	0.693	0.117	0.107	0.655	0.112	0.102
0.2	-1.61	0.00	0.45	0.005	0.003	-0.006	0.401	0.114	0.145	0.382	0.113	0.137
0.5	-0.69	0.00	0.71	0.003	0.001	-0.018	0.113	0.150	0.204	0.108	0.150	0.196
0.8	-0.22	0.00	0.89	-0.025	0.006	-0.025	0.297	0.185	0.318	0.283	0.178	0.310

Table S9 Summary of simulation results under Simulation setup III with censoring rate=28%

t	TRUE			Induced smoothing method								
				EB			ESE			ASE		
	$\alpha_0(t)$	$\alpha_1(t)$	$\beta(t)$	$\alpha_0(t)$	$\alpha_1(t)$	$\beta(t)$	$\alpha_0(t)$	$\alpha_1(t)$	$\beta(t)$	$\alpha_0(t)$	$\alpha_1(t)$	$\beta(t)$
$\tau = 0.5$												
0.1	-2.30	0.00	1.12	-0.055	-0.005	0.037	0.683	0.120	0.375	0.652	0.116	0.359
0.2	-1.61	0.00	0.64	-0.006	0.003	0.000	0.391	0.116	0.204	0.378	0.115	0.196
0.5	-0.69	0.00	0.43	0.004	0.001	-0.009	0.111	0.150	0.122	0.107	0.150	0.117
0.8	-0.22	0.00	0.45	-0.032	0.007	-0.014	0.294	0.185	0.159	0.278	0.179	0.155

Regularity conditions

We assume the following regularity conditions to establish the asymptotic properties of the proposed induced smoothed estimator. These conditions are similar to those imposed in Li *et al.* [1] and Kim *et al.* [2].

C1 There exists a finite positive constant M_c such that
- (a) $\sup_i \|\mathbf{W_i}\| \leq M_c$ where $\|\cdot\|$ denotes the Euclidean norm.
- (b) $\sup_{t\in\Gamma} |f_l(t)| \leq M_c$, $l = 0, 1, ..., L$.
- (c) $\sup_i \|\mathbf{Z_i(t)}\| \leq M_c$.

C2 The visit indicator η_{ij} is independent of T_i given $I(Y_i \geq t_{ij})$ and $\boldsymbol{W_i}$.

C3 There exists positive constant $\nu \geq 0$ such that
- (a) $P(C \geq \nu) = 0$ and $P(C = \nu) \geq c_0$, where c_0 is some positive constant.
- (b) $\sup_{\boldsymbol{Z}(t_{ij}),\boldsymbol{W},t_{ij}\in\Gamma}[t_{ij} + \exp\{\boldsymbol{\alpha}(t_{ij})^\top \mathbf{W} + \boldsymbol{\beta}(t_{ij})^\top \mathbf{Z}(t_{ij})\}] \leq \nu$.

C4 (a) For each $i = 1, ..., n$, $j = 1, ..., m$, $n^{-1}\sum_{i=1}^n \sum_{j=1}^m \boldsymbol{U}_i(t_{ij})\boldsymbol{U}_i(t_{ij})^\top E\{\eta_{ij}|I(T_i \geq t_{ij})\} g_{T_i-t_{ij}}(0)$ converges to a positive definite matrix $\boldsymbol{A}$.
- (b) For any $t_{ij} \in \Gamma$, the conditional density of $T_i - t_{ij}$ given $T \geq t_{ij}$, $g_{T_i-t_{ij}}\{s|T_i \geq t_{ij}\}$ is uniformly bounded away from 0 and from above. Also, $g'_{T_i-t_{ij}}(s|T_i \geq t_{ij})$ exists and is uniformly bounded on the real line.

Asymptotic properties of $\tilde{\gamma}$

The proofs follow from the asymptotic properties of the nonsmooth estimator $\boldsymbol{\gamma}$ and the arguments used in Kim *et al.* [2]. We first prove the consistency of $\tilde{\boldsymbol{\gamma}}$. Under C1-C4, based on the consistency result of $\hat{\boldsymbol{\gamma}}$ for $\boldsymbol{\gamma_0}$ [1], establishing the following convergence result is sufficient to prove the consistency of $\tilde{\boldsymbol{\gamma}}$: As $n \to \infty$

$$\sup_{\|\boldsymbol{\gamma}-\boldsymbol{\gamma_0}\|\leq\epsilon_n} \|n^{1/2}\{\tilde{\boldsymbol{S}}(\boldsymbol{\gamma};\tau,\boldsymbol{H}) - \boldsymbol{S}(\boldsymbol{\gamma};\tau)\}\| \xrightarrow{p} 0$$

where ϵ_n denotes a positive sequence converging to 0.

Let $\sigma_{ij} = \left\{\boldsymbol{U}_i(t_{ij})^\top \boldsymbol{H} \boldsymbol{U}_i(t_{ij})\right\}^{1/2}$, $\epsilon^{\gamma_{ij}} = \log(Y_i - t_{ij}) - \boldsymbol{\gamma}^\top \boldsymbol{U}_i(t_{ij})$ and $d_{ij}(\boldsymbol{\gamma}) = \mathrm{sgn}(\epsilon_{ij}{}^{\gamma})\Phi(-|\epsilon_{ij}{}^{\gamma}/\sigma_{ij}|)$, where sgn$(\cdot)$ is the sign function. Then,

$$
\begin{aligned}
& n^{1/2}\left\{\tilde{\boldsymbol{S}}(\boldsymbol{\gamma};\tau,\boldsymbol{H}) - \boldsymbol{S}(\boldsymbol{\gamma};\tau)\right\} \\
& = n^{-1/2}\sum_{i=1}^{n}\sum_{j=1}^{D}\eta_{ij} I(Y_i > t_{ij})\boldsymbol{U}_i(t_{ij})\frac{\hat{G}(t_{ij})\delta_i}{\hat{G}(Y_i)}\left\{\Phi\left(\frac{-\epsilon_{ij}^{\gamma}}{\sigma_{ij}}\right) - I[\epsilon_{ij}^{\gamma} < 0]\right\} \\
& = n^{-1/2}\sum_{i=1}^{n}\sum_{j=1}^{D}\eta_{ij} I(Y_i > t_{ij})\boldsymbol{U}_i(t_{ij})\delta_i\frac{G(t_{ij})}{G(Y_i)} d_{ij}(\boldsymbol{\gamma}) \\
& +\ n^{-1/2}\sum_{i=1}^{n}\sum_{j=1}^{D}\eta_{ij} I(Y_i > t_{ij})\boldsymbol{U}_i(t_{ij})\delta_{ij}\left\{\frac{\hat{G}(t_{ij})}{\hat{G}(Y_i)} - \frac{G(t_{ij})}{G(Y_i)}\right\} d_{ij}(\boldsymbol{\gamma}) \\
& = \boldsymbol{S}^{D(1)}(\boldsymbol{\gamma};\tau,\boldsymbol{H}) + \boldsymbol{S}^{D(2)}(\boldsymbol{\gamma};\tau,\boldsymbol{H}).
\end{aligned}
$$

For a specific $t_{ij} \in \Gamma$, by Condition C2, we have

$$
E\{\boldsymbol{S}^{D(1)}(\boldsymbol{\gamma};\tau,\boldsymbol{H})\} = n^{-1/2}\sum_{i=1}^{n}\sum_{j=1}^{D}\eta_{ij}\boldsymbol{U}_i(t_{ij})E\left\{\eta_{ij}|I(T_i > t_{ij})\right\}E\{d_{ij}(\boldsymbol{\gamma})|I(T_i > t_{ij})\}.
$$

Note that

$$
\begin{aligned}
E\{d_{ij}(\boldsymbol{\gamma})|I(T_i > t_{ij})\} &= \int_{-\infty}^{\infty}\mathrm{sgn}(\epsilon_{ij}^{\gamma})\Phi\left(-\left|\frac{\epsilon_{ij}{}^{\gamma}}{\sigma_{ij}}\right|\right)g_{T_i - t_{ij}}\{\epsilon_{ij}^{\gamma} + \boldsymbol{U}_i(t_{ij})^\top(\boldsymbol{\gamma} - \boldsymbol{\gamma_0})\}d\epsilon_{ij}^{\gamma} \\
&= \sigma_{ij}\int_{-\infty}^{\infty}\Phi\left(-|t_{ij}|\right)\left\{2I(t) > 0) - 1\right\}g_{T_i - t_{ij}}\left\{\sigma_{ij}t + \boldsymbol{U}_i(t_{ij})^\top(\boldsymbol{\gamma} - \boldsymbol{\gamma_0})\right\}dt \\
&= \sigma_{ij}\int_{-\infty}^{\infty}\Phi\left(-|t|\right)\left\{2I(t) > 0) - 1\right\}[g_{T_i - t_{ij}}\left\{\boldsymbol{U}_i(t_{ij})^\top(\boldsymbol{\gamma} - \boldsymbol{\gamma_0})\right\} \\
&+ g'_{T_i - t_{ij}}\{w^*{}_i(t_{ij})\}\sigma_{ij}t]dt
\end{aligned}
$$

where $w_i^*(t_{ij})$ is the line segment that lies between $\boldsymbol{U}_i(t_{ij})^\top(\boldsymbol{\gamma} - \boldsymbol{\gamma}_0)$ and $\boldsymbol{U}_i(t_{ij})^\top(\boldsymbol{\gamma} - \boldsymbol{\gamma}_0) + \sigma_{ij}t_{ij}$. By Condition C4(b), we have $\sup_{i,j} g_{T_i - t_{ij}}\{\boldsymbol{U}_i(t_{ij})^\top(\boldsymbol{\gamma} - \boldsymbol{\gamma_0})\} < \infty$. Then, it follows that $\int_{-\infty}^{\infty}\Phi(-|t|)\{2I(t > 0) - 1\}g_{T_i - t_{ij}}\{\boldsymbol{U}_i(t_{ij})^\top(\boldsymbol{\gamma} - \boldsymbol{\gamma}_0)\}dt = 0$ since $\int_{-\infty}^{\infty}\Phi(-|t|)\{2I(t > 0) - 1\}dt = 0$. By Condition C4 (b), we can also identify $\exists M > 0$ such that $\sup_{i,j}|g'_{T_i - t_{ij}}\{w^*{}_{ij}(t_{ij})\}| < M$. Then, by combining the fact that $\int_{-\infty}^{\infty}|t|\Phi(-|t|)dt = 1/2$, we have

$$
|E\{d_{ij}(\boldsymbol{\gamma})|I(T_i > t_{ij})\}| \le \sigma_{ij}^2\int_{-\infty}^{\infty}|t|\Phi(-|t|)|g'_{\gamma,i,j}\{w_{ij}^*(t_{ij})\}|dt_{ij} \le M\sigma_{ij}^2/2.
$$

Note that $\sum_{i=1}^{n}\sum_{j=1}^{D}\sigma_{ij}{}^2 = \mathrm{tr}(\boldsymbol{U}_i(t_{ij})\boldsymbol{H}\boldsymbol{U}_i(t_{ij})^\top) = \mathrm{tr}(\boldsymbol{H}\boldsymbol{U}_i(t_{ij})^\top\boldsymbol{U}_i(t_{ij}))$ is bounded from Conditions C1, C4 (b) and the fact that $\boldsymbol{H} = O(n^{-1})$. In turn,

$\sum_{i=1}^{n}\sum_{j=1}^{D}|E\{d_{ij}(\boldsymbol{\gamma})|I(T_i > t_{ij})\}| \le M\sum_{i=1}^{n}\sum_{j=1}^{D}{\sigma_{ij}}^2/2$ is also bounded. Therefore, as $n \to \infty$,

$$\|E\{\boldsymbol{S}^{D(1)}(\boldsymbol{\gamma};\tau,\boldsymbol{H})\}\| \le n^{-1/2}\sqrt{p}\sup_{i,j}|\boldsymbol{U}_i(t_{ij})|\sum_{i=1}^{n}\sum_{j=1}^{n}|E\{d_{ij}(\boldsymbol{\gamma})|I(T_i > t_{ij})\}| \to 0. \tag{1}$$

For $\text{Var}\{\boldsymbol{S}^{D(1)}(\boldsymbol{\gamma};\tau,\boldsymbol{H})\}$, by Conditions C2 and C3, we have

$$\begin{aligned}\text{Var}\{\boldsymbol{S}^{D(1)}(\boldsymbol{\gamma};\tau,\boldsymbol{H})\} &= \text{Var}\left\{\frac{1}{n}\sum_{i=1}^{n}\sum_{j=1}^{D}\eta_{ij}I(Y_i > t_{ij})\boldsymbol{U}_i(t_{ij})\boldsymbol{U}_i(t_{ij})^\top\frac{\delta_i G(t_{ij})}{G(Y_i)}d_{ij}(\boldsymbol{\gamma})\right\}\\ &\le \frac{1}{n}\sum_{i=1}^{n}\sum_{j=1}^{D}\frac{\boldsymbol{U}_i(t_{ij})\boldsymbol{U}_i(t_{ij})^\top}{\nu}E\left\{\eta_{ij}|I(T_i > t_{ij})\right\}E\{d_{ij}^2(\boldsymbol{\gamma})|I(T_i > t_{ij})\}.\end{aligned}$$

where

$$\begin{aligned}&E\{d_{ij}^2(\boldsymbol{\gamma})|I(T_i > t_{ij})\}\\ &= \int_{-\infty}^{\infty}\Phi^2(-|s|)g_{T_i-t_{ij}}\{\sigma_{ij}s + \boldsymbol{U}_i(t_{ij})^\top(\boldsymbol{\gamma}-\boldsymbol{\gamma_0})\}d(\sigma_{ij}s)\\ &= \int_{|s|>\Delta}\Phi^2(-|s|)g_{T_i-t_{ij}}\{\sigma_{ij}s + \boldsymbol{U}_i(t_{ij})^\top(\boldsymbol{\gamma}-\boldsymbol{\gamma_0})\}d(\sigma_{ij}s)\\ &+ \int_{|s|\le\Delta}\Phi^2(-|s|)g_{T_i-t_{ij}}\{\sigma_{ij}s + \boldsymbol{U}_i(t_{ij})^\top(\boldsymbol{\gamma}-\boldsymbol{\gamma_0})\}d(\sigma_{ij}s)\\ &\le \Phi^2(-\Delta) + \sigma_{ij}\int_{|s|\le\Delta}g_{T_i-t_{ij}}\{\sigma_{ij}s + \boldsymbol{U}_i(t_{ij})^\top(\boldsymbol{\gamma}-\boldsymbol{\gamma_0})\}ds\\ &= \Phi^2(-\Delta) + 2\sigma_{ij}\Delta g_{T_i-t_{ij}}({w_{ij}}^*).\end{aligned}$$

Let $\Delta = n^{1/4}$. Then, as $n \to \infty$, $\sigma_{ij}\Delta \to 0$ since $\sigma_{ij} = O(n^{-1/2})$. It follows from applying Condition C4(b) that both $\Phi^2(-\Delta)$ and $\sigma_{ij}\Delta g_{T_i-t_{ij}}({w_{ij}}^*)$ approach 0 as $n \to \infty$. Therefore, as $n \to \infty$, $\|E\{{d_{ij}}^2(\boldsymbol{\gamma})|I(T_i > t_{ij})\}\| \to 0$.

This implies, as $n \to \infty$,

$$\|\text{Var}\{\boldsymbol{S}^{D(1)}(\boldsymbol{\gamma};\tau,\boldsymbol{H})\}\| \to 0.$$

By the Weak Law of Large Numbers, for $\boldsymbol{\gamma}$ satisfying $\|\boldsymbol{\gamma}-\boldsymbol{\gamma_0}\| \le \varepsilon_n$, we have

$$\|\boldsymbol{S}^{D(1)}(\boldsymbol{\gamma})\| \xrightarrow{p} 0, \qquad \text{as } n \to \infty. \tag{2}$$

In showing $\|\boldsymbol{S}^{D(2)}(\boldsymbol{\gamma})\| \xrightarrow{p} 0$ as $n \to \infty$, we require the following Martingale representation of the Kaplan-Meier estimator [3] for $\hat{G}(t)$:

$$\frac{\hat{G}(t)-G(t)}{G(t)} = -\sum_{i=1}^{n}\int_0^t\left\{\frac{\hat{G}(u^-)}{G(u)}\right\}\frac{d{M_i}^c(u)}{Y(u)}$$

where

$$M_i^c(u) = N_i^c(u) - \int_0^t I(Y_i \geq u) d\Lambda^c(s), N_i^c(u) = (1-\delta_i) I(Y_i \leq u), \Lambda^c(u) \quad = -\log\{G(u)\}, \text{ and}$$

$$Y(u) = \sum_{i=1}^n I(Y_i \geq u).$$

In combination with an application of the functional delta method, uniform convergences of $\hat{G}(\cdot)$ to $G(\cdot)$, and $Y(u)$ to $y(u) = \lim_{n\to\infty} n^{-1}\sum_{i=1}^n I(Y_i \geq u)$, it can be shown that

$$\boldsymbol{S}^{D(2)}(\boldsymbol{\gamma}) = n^{-1/2} \sum_{k=1}^n \int_0^L \left\{ n^{-1} \sum_{i=1}^n \sum_{j=1}^D \eta_{ij} I(Y_i \geq t_{ij}) \boldsymbol{U}_i(t_{ij}) \delta_i I(Y_i \geq u) \frac{G(t_{ij})}{G(Y_i)} d_{ij}(\boldsymbol{\gamma}) \right\} \frac{dM_k^c(u)}{y(u)} + o_p(1).$$

Denote

$$\mathbb{I}_n(u, \boldsymbol{\gamma}) = n^{-1} \sum_{i=1}^n \sum_{j=1}^D \eta_{ij} I(Y_i \geq t_{ij}) U_i(t_{ij}) \delta_i I(Y_i \geq u) \frac{G(t_{ij})}{G(Y_i)} d_{ij}(\boldsymbol{\gamma}).$$

Then,

$$E\{\mathbb{I}_n(u,\boldsymbol{\gamma})\} = n^{-1} \sum_{i=1}^n \sum_{j=1}^D \boldsymbol{U}_i(t_{ij}) E\left\{\eta_{ij} | I(T_i > t_{ij})\right\} E\{I(Y_i \geq u) d_{ij}(\boldsymbol{\gamma}) | I(T_i \geq t_{ij})\}.$$

Since $|E\{I(Y_i \geq u) d_{ij}(\boldsymbol{\gamma}) | I(T_i \geq t_{ij})\}| \leq E\{|d_{ij}(\boldsymbol{\gamma})| | I(T_i \geq t_{ij})\}$ and it can be shown $E\{|d_{ij}(\boldsymbol{\gamma})| | I(T_i \geq t_{ij})\} = O(n^{-1/2})$,

$$\|E\{\mathbb{I}_n(u,\boldsymbol{\gamma})\}\| \leq \sqrt{p} \sup_{i,j} |\boldsymbol{U}_i(t_{ij})| n^{-1} \sum_{i=1}^n \sum_{j=1}^D \eta_{ij} E\{|d_{ij}(\boldsymbol{\gamma})| | I(T_i \geq t_{ij})\} \to 0 \text{ as } n \to \infty.$$

It follows from the Martingale central limit theorem [3] and the Kolmogorov-Centsov Theorem [4, p53] that $n^{-1/2}\sum_{k=1}^n \dfrac{dM_k^c(u)}{y(u)}$ converges weakly to a zero-mean Gaussian process with continuous sample paths. Then, for $\boldsymbol{\gamma}$ satisfying $\|\boldsymbol{\gamma} - \boldsymbol{\gamma_0}\| \leq \varepsilon_n$, we have

$$\left\| n^{-1/2} \sum_{k=1}^n \int_0^L \mathbb{I}_n(u,\boldsymbol{\gamma}) \frac{d{M_k}^c(u)}{y(u)} \right\| \xrightarrow{p} 0 \text{ as } n \to \infty \tag{3}$$

by Lemma 1 in [5].

From (2) and (3), we have

$$\sup_{\|\boldsymbol{\gamma}-\boldsymbol{\gamma_0}\| \leq \epsilon_n} \|n^{1/2}\{\tilde{\boldsymbol{S}}(\boldsymbol{\gamma};\tau,\boldsymbol{H}) - \boldsymbol{S}(\boldsymbol{\gamma};\tau)\}\| \xrightarrow{p} 0 \text{ as } n \to \infty \tag{4}$$

This point-wise convergence result can be strengthened to uniform convergence since $\tilde{\boldsymbol{S}}_n(\boldsymbol{\gamma};\tau,\boldsymbol{H})$ and $\boldsymbol{S}_n(\boldsymbol{\gamma};\tau)$ are monotonic functions [6].

To establish the asymptotic normality of $n^{1/2}(\tilde{\boldsymbol{\gamma}} - \boldsymbol{\gamma_0})$, we show the asymptotic equivalence between $n^{1/2}(\tilde{\boldsymbol{\gamma}}-\boldsymbol{\gamma_0})$ and $n^{1/2}(\hat{\boldsymbol{\gamma}}-\boldsymbol{\gamma_0})$. The consistency and the asymptotic normality of $\hat{\boldsymbol{\gamma}}$, the nonsmoothed method estimator, has been established in Li *et al.* [1]. Thus, combining these results with the convergence result in (4), it suffices to show the following convergence result [2, 7]: As $n \to \infty$, for any positive definite matrix $\boldsymbol{H}$,

$$\|\tilde{\boldsymbol{A}}_n(\boldsymbol{\gamma_0}) - \boldsymbol{A}\| \xrightarrow{p} 0. \tag{5}$$

Let $a, b \in \boldsymbol{R}^p$. Then,

$$E\left[\tilde{\boldsymbol{A}}_n(\boldsymbol{\gamma_0})\right] = a^\top \left[n^{-1}\sum_{i=1}^{n}\sum_{j=1}^{D}\boldsymbol{U}_i(t_{ij})\boldsymbol{U}_i(t_{ij})^\top E\{\eta_{ij}|I(T_i \geq t_{ij})\}E\left\{\phi\left(-\frac{\epsilon_{ij}}{\sigma_{ij}}\right)\frac{1}{\sigma_{ij}}\middle| I(T_i \geq t_{ij})\right\}\right] b.$$

$$\begin{aligned} E\left\{\phi\left(-\frac{\epsilon_{ij}}{\sigma_{ij}}\right)\frac{1}{\sigma_{ij}}\middle| I(T_i \geq t_{ij})\right\} &= \frac{1}{\sigma_{ij}}\int_{-\infty}^{\infty}\phi\left(-\frac{\epsilon_{ij}}{\sigma_{ij}}\right)g_{T_i-t_{ij}}(\epsilon_{ij})d\epsilon_{ij} = \int_{-\infty}^{\infty}\phi(-t)g_{T_i-t_{ij}}(\sigma_{ij}t)dt \\ &= \int_{-\infty}^{\infty}\phi(-t)g_{T_i-t_{ij}}(0)dt + \sigma_{ij}\int_{-\infty}^{\infty}t\phi(-t)g'_{T_i-t_{ij}}(\omega^*)dt, \end{aligned}$$

where ω^* is the line segment lying between 0 and $\sigma_{ij}t$. By applying Condition C4 (b), the second term on the right-hand side of the equation above can be shown to converge to 0, i.e., $\exists M > 0$,

$$\sigma_{ij}\int_{-\infty}^{\infty}t\phi(-t)g'_{T_i-t_{ij}}(\omega^*)dt \leq M\sigma_{ij}\int_{-\infty}^{\infty}|t|\phi(-t)dt \xrightarrow{p} 0.$$

Then, it follows from $\int_{-\infty}^{\infty}\phi(-t)g_{T_i-t_{ij}}(0)dt = g_{T_i-t_{ij}}(0)$ and Condition 4 (a) that $E\left\{\phi\left(-\frac{\epsilon_{ij}}{\sigma_{ij}}\right)\frac{1}{\sigma_{ij}}\middle| I(T_i \geq t_{ij})\right\} \xrightarrow{p} g_{T_i-t_{ij}}(0)$ and

$$\begin{aligned} &\lim_{n\to\infty} E\left[a^\top\left\{n^{-1}\sum_{i=1}^{n}\sum_{j=1}^{D}\eta_{ij}I(Y_i > t_{ij})\boldsymbol{U}_i(t_{ij})\boldsymbol{U}_i(t_{ij})^\top\frac{\hat{G}(t_{ij})\delta_i}{\hat{G}(Y_i)}\phi\left(-\frac{\epsilon_{ij}}{\sigma_{ij}}\right)\frac{1}{\sigma_{ij}}\right\}b\right] \\ &= a^\top\left\{\lim_{n\to\infty} n^{-1}\sum_{i=1}^{n}\sum_{j=1}^{D}\boldsymbol{U}_i(t_{ij})\boldsymbol{U}_i(t_{ij})^\top E\{\eta_{ij}|I(T_i > t_{ij})\}g_{T_i-t_{ij}}(0)\right\}b \\ &= a^\top\boldsymbol{A}b. \end{aligned} \tag{6}$$

Now, applying the same arguments used for $E\left\{\phi\left(-\frac{\epsilon_{ij}}{\sigma_{ij}}\right)\frac{1}{\sigma_{ij}}\middle| I(T_i \geq t_{ij})\right\}$, it can be shown that

$$\begin{aligned}
&E\left[\left\{\phi\left(-\frac{\epsilon_{ij}}{\sigma_{ij}}\right)\frac{1}{\sigma_{ij}}\right\}^2\middle| I(T_i \geq t_{ij}), \boldsymbol{U}_i(t_{ij})\right] \\
&= \frac{1}{\sigma_{ij}^2}\int_{-\infty}^{\infty}\phi^2\left(-\frac{\epsilon_{ij}}{\sigma_{ij}}\right) g_{T_i - t_{ij}}(\epsilon_{ij}) d\epsilon_{ij} \\
&= \frac{1}{\sigma_{ij}}\int_{-\infty}^{\infty}\phi^2(-t) g_{T_i - t_{ij}}(0) dt + \int_{-\infty}^{\infty} t\phi^2(-t) g'_{T_i - t_{ij}}(\omega^*) dt \\
&= O(n^{1/2}).
\end{aligned}$$

Consequently,

$$\begin{aligned}
&\text{Var}\left[a^\top\left\{n^{-1}\sum_{i=1}^{n}\sum_{j=1}^{D}\eta_{ij} I(Y_i > t_{ij})\boldsymbol{U}_i(t_{ij})\boldsymbol{U}_i(t_{ij})^\top\frac{G(t_{ij})\delta_i}{G(Y_i)}\phi\left(-\frac{\epsilon_{ij}}{\sigma_{ij}}\right)\frac{1}{\sigma_{ij}}\right\} b\right] \\
&\leq \frac{1}{n^2\nu}\sum_{i=1}^{n}\sum_{j=1}^{D}(a^\top\boldsymbol{U}_i(t_{ij})\boldsymbol{U}_i(t_{ij})^\top b)^2 E\{\eta_{ij}|I(T_i \geq t_{ij})\} E\left[\left\{\phi\left(-\frac{\epsilon_{ij}}{\sigma_{ij}}\right)\frac{1}{\sigma_{ij}}\right\}^2\middle| T_i \geq t_{ij}, \boldsymbol{U}_i(t_{ij})\right] \to 0.
\end{aligned} \tag{7}$$

From (6) and (7), we have the desired result: $\|\tilde{\boldsymbol{A}}_n(\boldsymbol{\gamma_0}) - \boldsymbol{A}\| \xrightarrow{p} 0$ as $n \to \infty$.

Note that the asymptotic covariance function is the same as that of the nonsmooth estimator. Its explicit form is given as

$$\lim_{n\to\infty}\text{Var}\left[n^{-1/2}\sum_{i=1}^{n} -P^{-1}(\boldsymbol{\gamma})\left\{\psi_i^G(\boldsymbol{\gamma}) + \zeta_i(\boldsymbol{\gamma})\right\}\right]$$

where

$$\begin{aligned}
P(\boldsymbol{\gamma}) &= \partial\mu(\boldsymbol{\gamma})/\partial\boldsymbol{\gamma}, \mu(\boldsymbol{\gamma}) = E\left\{\psi_i^G(\boldsymbol{\gamma})\right\}, \psi_i^G(\boldsymbol{\gamma}) = \sum_{j=1}^{D}\eta_{ij}\boldsymbol{S}_i^G(\boldsymbol{\gamma}, t_j), \\
\boldsymbol{S}_i^G(\boldsymbol{\gamma}, t) &= I(Y_i > t)\boldsymbol{U}_i(t)\left[\frac{I\{(\log(Y_i - t) \leq \boldsymbol{\gamma}^\top\boldsymbol{U}_i(t), \delta_i = 1\}}{G(T_i)/G(t)} - \tau\right], \\
\zeta_k(\boldsymbol{\gamma}) &= \mathrm{E}\left[\sum_{j=1}^{D}\boldsymbol{V}_{ij}(\boldsymbol{\gamma})\left\{\frac{g_k(t_j)}{G(T_i)} - \frac{g_k(T_i)G(t_j)}{G^2(T_i)}\right\}\middle|\mathbb{D}_k\right], \text{ and} \\
\boldsymbol{V}_{ij}(\boldsymbol{\gamma}) &= \eta_{ij} I(Y_i > t_j)\boldsymbol{U}_i(t_j) I\{\log(Y_i - t_j) \leq \boldsymbol{\gamma}^\top\boldsymbol{U}_i(t_j), \delta_i = 1\}.
\end{aligned}$$

Here $\mathbb{D}_k$ denote the observed data from the kth subject. The Kaplan-Meier estimator $\hat{G}(\cdot)$ of $G(\cdot)$ is asymptotically represented as $\hat{G}(t) - G(t) = n^{-1}\sum_{i=1}^{n} g_i(t) + o_p(n^{-1/2})$ where the analytic form of $g_i(t)$ can be found in Reid [8].

Author details

References

1. Li, R., Huang, X., Cortes, J.: Quantile residual life regression with longitudinal biomarker measurements for dynamic prediction. Journal of the Royal Statistical Society. Series C (Applied Statistics), 755–773 (2016)

2. Kim, K.H., Caplan, D.J., Kang, S.: Smoothed quantile regression for censored residual life. Computational Statistics (2022). doi:10.1007/s00180-022-01262-z
3. Fleming, T.R., Harrington, D.P.: Counting Processes and Survival Analysis. John Wiley & Sons, New Jersey (2011)
4. Karatzas, I., Shreve, S.E.: Brownian Motion and Stochastic Calculus vol. 113. Springer, New York (1991)
5. Lin, D.: On fitting cox's proportional hazards models to survey data. Biometrika **87**(1), 37–47 (2000)
6. Shorack, G.R., Wellner, J.A.: Empirical Processes with Applications to Statistics. SIAM, Philadelphia (2009)
7. Pang, L., Lu, W., Wang, H.J.: Variance estimation in censored quantile regression via induced smoothing. Computational statistics & data analysis **56**(4), 785–796 (2012)
8. Reid, N.: Influence Functions for Censored Data. The Annals of Statistics **9**(1), 78–92 (1981). doi:10.1214/aos/1176345334
